# Mechanistic and therapeutic perspectives of non-coding RNA-modulated apoptotic signaling in diabetic retinopathy

**DOI:** 10.1007/s10565-024-09896-z

**Published:** 2024-07-06

**Authors:** Qin Wu, Chunlei Liu, Xiangwen Shu, Lian Duan

**Affiliations:** 1 Jinan Second People’s Hospital & The Ophthalmologic Hospital of Jinan, Jinan, 250021 China; 2 Aier Eye Hospital, Liaocheng, 252005 China; 3 Department of Ophthalmology, The First Affiliated Hospital of Shandong First Medical University & Shandong Provincial Qianfoshan Hospital, Jinan, 250014 China

**Keywords:** Diabetic retinopathy, Apoptosis, ncRNA

## Abstract

Diabetic retinopathy (DR), a significant and vision-endangering complication associated with diabetes mellitus, constitutes a substantial portion of acquired instances of preventable blindness. The progression of DR appears to prominently feature the loss of retinal cells, encompassing neural retinal cells, pericytes, and endothelial cells. Therefore, mitigating the apoptosis of retinal cells in DR could potentially enhance the therapeutic approach for managing the condition by suppressing retinal vascular leakage. Recent advancements have highlighted the crucial regulatory roles played by non-coding RNAs (ncRNAs) in diverse biological processes. Recent advancements have highlighted that non-coding RNAs (ncRNAs), including microRNAs (miRNAs), circular RNAs (circRNAs), and long non-coding RNAs (lncRNAs), act as central regulators in a wide array of biogenesis and biological functions, exerting control over gene expression associated with histogenesis and cellular differentiation within ocular tissues. Abnormal expression and activity of ncRNAs has been linked to the regulation of diverse cellular functions such as apoptosis, and proliferation. This implies a potential involvement of ncRNAs in the development of DR. Notably, ncRNAs and apoptosis exhibit reciprocal regulatory interactions, jointly influencing the destiny of retinal cells. Consequently, a thorough investigation into the complex relationship between apoptosis and ncRNAs is crucial for developing effective therapeutic and preventative strategies for DR. This review provides a fundamental comprehension of the apoptotic signaling pathways associated with DR. It then delves into the mutual relationship between apoptosis and ncRNAs in the context of DR pathogenesis. This study advances our understanding of the pathophysiology of DR and paves the way for the development of novel therapeutic strategies.

**Graphical Abstract:**

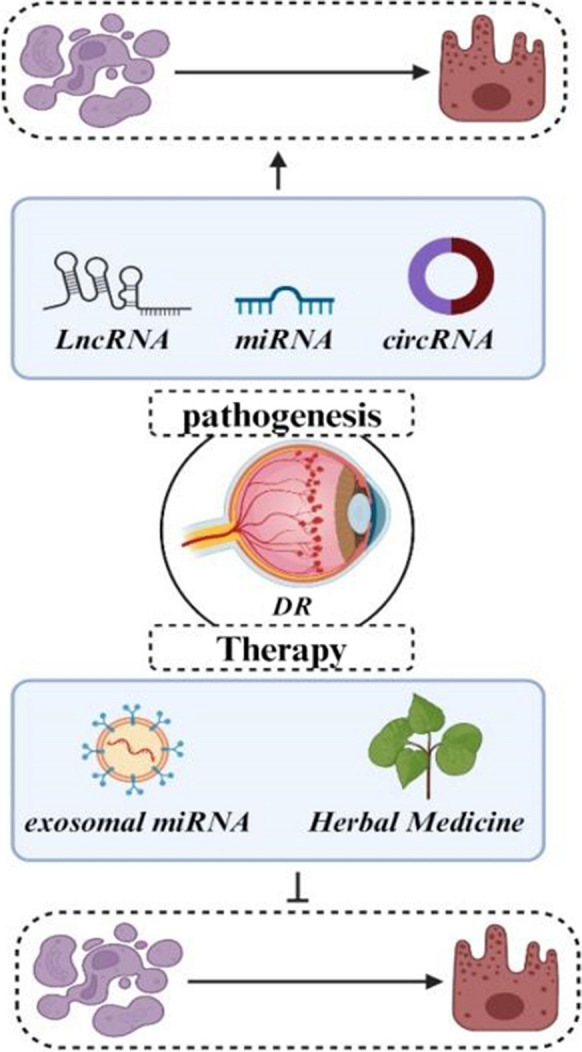

## Introduction

Diabetes mellitus is a prevalent global health disorder, with an estimated incidence of up to 285 million cases (Dhankhar et al. 2023). This condition is characterized by consistently high blood sugar levels due to insufficient insulin production by the pancreas or decreased sensitivity of cells to insulin. Type 1 diabetes, also known as insulin-dependent diabetes, is linked to inadequate insulin secretion, while Type 2 diabetes, known as insulin-independent diabetes, is associated with reduced cell responsiveness to insulin (Ozougwu et al. 2013). Hyperglycemia leads to various microvascular abnormalities in different parts of the body, including the retina, which can result in conditions like retinal detachment, neovascularization, and macular edema. These changes in the retina indicate damage caused by high blood sugar levels and the progression of diabetic retinopathy, a condition that significantly contributes to the risk of vision loss but can be preventable with appropriate interventions (Nentwich and Ulbig 2015).

The retina is a specialized tissue that plays a crucial role in light perception and the creation of visual images. It consists of various types of neuronal cell types, including bipolar cells, amacrine cells, retinal ganglion cells, cones, rods, and horizontal cells, as well as different glial cell types like microglia, astrocytes, and Müller cells (Grossniklaus et al. 2015; Masland 2012; Mahabadi and Khalili 2023). Apoptosis, a form of programmed cell death, is linked to the development of diabetic retinopathy (DR), where the loss of retinal cells is a significant feature of the condition's progression (Khalfaoui et al. 2010). Different types of retinal cells, such as endothelial cells, pericytes, neural retinal cells such as ganglion cells, and retinal glial cells like microglia, astrocytes, and Müller cells, have been observed to undergo various forms of cell death (Walshe et al. 2011; Santiago et al. 2007). Research has focused on understanding the apoptotic processes involving retinal cells during the disease's advancement. Studies suggest that endothelial cells primarily undergo apoptotic cell deathpossibly through a caspase-dependent mechanism involving mitochondrial dysfunction. However, further investigation is needed to explore whether this pathway is intrinsic or extrinsic in nature (Feenstra et al. 2013).

**Fig. 1:**
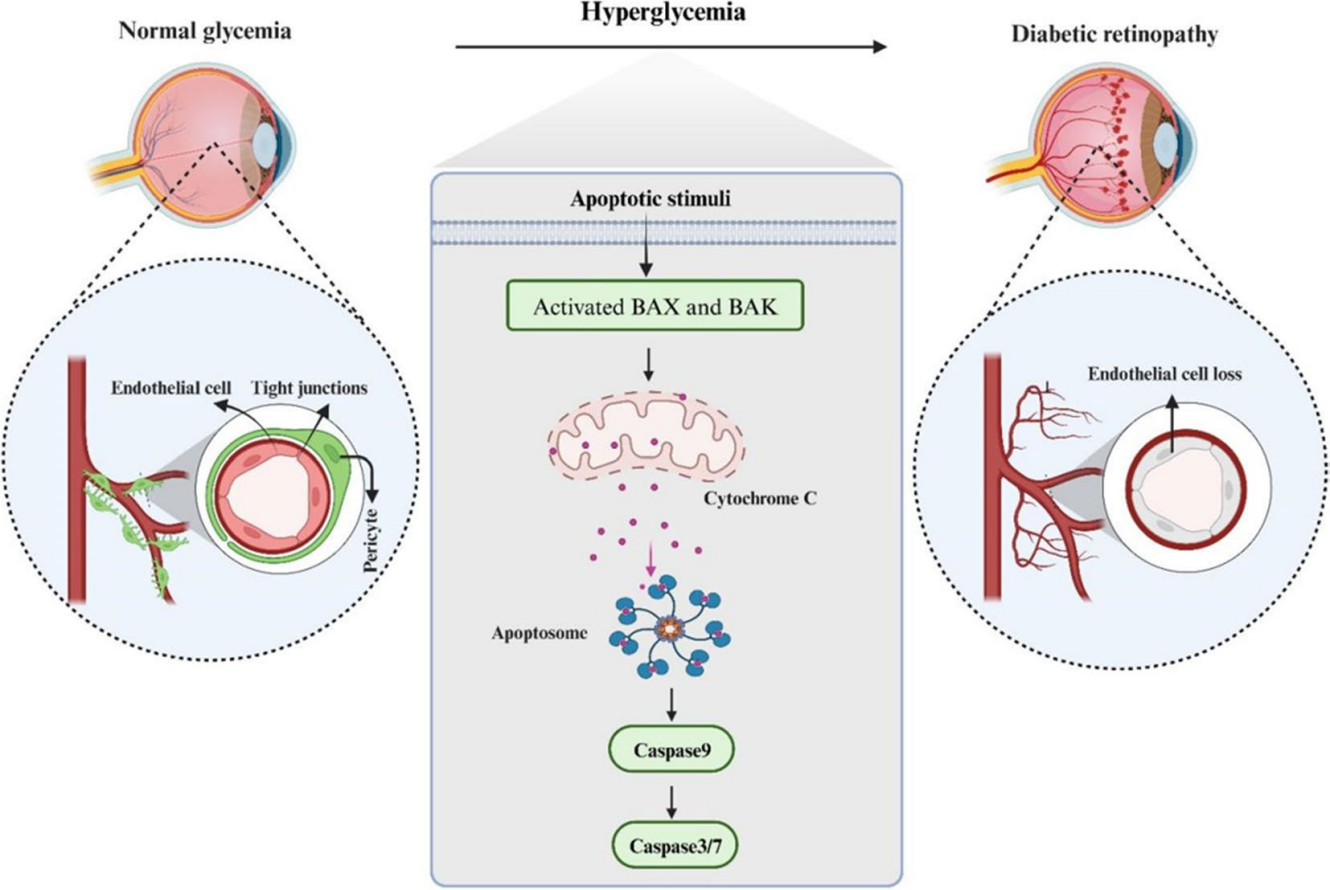
Schematic representation illustrating the pivotal role of apoptosis in the progression of diabetic retinopathy. This visual representation aims to provide a comprehensive overview of the apoptotic processes underlying DR progression, offering insights into potential therapeutic avenues for mitigating vision impairment in individuals with diabetes

ncRNAs, which include miRNAs, circRNAs, and lncRNAs, are essential in modulating various cellular processes through their functions as regulatory molecules (Esteller 2011; Hombach and Kretz 2016). These ncRNAs mediate cellular regulatory processes including post-transcriptional modifications, signal transduction, transcriptional regulation, and chromatin remodeling. Research results suggest that ncRNAs display distinct expression patterns in DR and contribute substantially to the onset and advancement of this condition. In this context, recent experimental studies have shown a significant increase in the expression of miR-211 in hyperglycemic HUVECs and in diabetic retinal tissues. This increase in expression significantly inhibits the activity of the downstream target gene, Sirtuin 1 (SIRT1), by binding specifically to the 3'-untranslated region (3'-UTR) of the gene. Consequently, this molecular mechanism contributes to the development of retinal vascular dysfunction and DR. Thus, miR-211 might engage in the pathophysiological processes observed in individuals with DR through its interaction with the target gene SIRT1 (Liu et al. 2018). Emerging research has demonstrated a significant role for ncRNAs in regulating apoptosis within retinal cells. In this context, Wan et al. explored the protective influence exerted by miR-200a during the progression of DR. They observed reduced levels of miR-200a and elevated LIM domain protein 1 (PDLIM1) levels in both in vivo and in vitro DR models. Administration of miR-200a effectively mitigated retinal permeability and the expression of inflammatory factors. Furthermore, by targeting PDLIM1, miR-200a was observed to alleviate apoptotic conditions, enhance cell viability, and markedly reduce cellular migration in high-glucose-treated HRMECs. So, miR-200a presents itself as a prospective therapeutic target for the modulation of PDLIM1 expression DR (Wan et al. 2021). Similarly, recent evidence has revealed a direct interaction between miR-29 and the lncRNA RNA VIM-AS1. Also, exposure to high glucose (HG) resulted in upregulated miR-29 expression and downregulated VIM-AS1 levels. Furthermore, examination of cellular apoptosis demonstrated that the increased expression of VIM-AS1 mitigated the pro-apoptotic impact of miR-29 overexpression on h1RPE7 cells under HG conditions. Therefore, VIM-AS1 functions as a potential competitor for miR-29 binding, thereby influencing apoptotic regulation within h1RPE7 cells and potentially contributing to the development of DR (Zeng et al. 2020a).. Thereby, in the current paper, we initially present a detailed explanation of the apoptosis signaling pathway within the framework of DR. Subsequently, we elucidate the influence of ncRNAs on cellular apoptosis within the DR framework. Finally, in the concluding section, we illustrate the potential therapeutic utility of targeting ncRNAs/apoptosis axis in the management of DR (Fig. 1) (Table 1).

**Table 1:** An overview of major non-coding RNAs in DR, and their influence on retinal cell apoptosis

NcRNA	Name	Target	Expression levels in HG condition or DR model	Cells or model	Role in apoptosis	Ref
miRNA	miR-122	TIMP3	Upregulation	ARPE-19	Promoting	Wang et al. 2020a)
	miR-200a-3p	TGF-β2	Downregulation	ARPE-19	Suppressing	Xue et al. 2020)
	miR-200b	Oxr1	Upregulation	MIO-M1	Promoting	Murray et al. 2013)
	miR-423-5p	TFF1	Upregulation	RPE	Promoting	Xiao et al. 2019)
	miR-455-5p	SOCS3	Downregulation	RPE	Suppressing	Chen et al. 2019)
	miR-29a	-	Downregulation	HLE B-3	Suppressing	Li et al. 2021b)
	miR-29	PTEN	Upregulation	ARPE-19	Promoting	Lin et al. 2016)
	miR-29b-3p	SIRT1	Upregulation	HRMECs	Promoting	Zeng et al. 2020b)
	miR-204	-	Downregulation	Rat	Suppressing	Qi et al. 2020)
	miR-211	SIRT1	Upregulation	Mice	Promoting	Zeng et al. 2017a)
	miR-365	IGF-1	Upregulation	Retinal neurons	Promoting	Zheng et al. 2018)
	miR-126	IL-17A	Downregulation	HRECs	Suppressing	Chen et al. 2020a)
	miR-21	PPARα	Upregulation	Mice	Promoting	Chen et al. 2017a)
	miR-146a	IL-6	Downregulation	REC	Suppressing	Ye and Steinle 2017)
	miR-219-5p	LRH-1	Upregulation	ARPE-19	Promoting	Zhao et al. 2018)
	miR-495	Notch1	Upregulation	RGCs	Promoting	Zhang et al. 2018)
	miR-383	PRDX3	Upregulation	ARPE-19	Promoting	Jiang et al. 2017)
	miR-218	RUNX2	Upregulation	ARPE-19	Promoting	Yao et al. 2019)
	miR-542-5p	CARM1	Downregulation	RPE	Suppressing	Guo et al. 2020)
	miR-133b	RhoA	Downregulation	REC	Promoting	Yao et al. 2018)
	miR-499-3p	IFNA2	Upregulation	Rat	Promoting	Liu et al. 2020)
	miR-34a	SIRT1	Upregulation	RVECs	Promoting	Ji et al. 2020)
		HMGB1	Upregulation	Rat	Promoting	Ma et al. 2020)
	miR-19a	PTEN	Upregulation	Rat	Promoting	Zhang and Liu 2020)
	miR-203a-3p	SOCS3	Upregulation	Rat	Promoting	Zhang et al. 2020)
	miR-296-5p	GNAI2	Downregulation	Mice	Suppressing	Che et al. 2022)
	miR-345-5p	Atf1	Upregulation	RGCs	Promoting	Ge et al. 2021)
	miR-221	SIRT1	Upregulation	hRMEC	Promoting	Chen et al. 2020b)
lncRNA	lncRNA MEG3	Induce SOCS6, by sponging miR-19b	Downregulation	hRMECs	Suppressing	Xiao et al. 2020)
		Induce Nrf2, by sponging miR-93	Downregulation	RPE	Suppressing	Luo et al. 2020)
		Induce SIRT1, by sponging miR-34a	Downregulation	ARPE-19	Suppressing	Tong et al. 2019)
	lncRNA PVT1	Induce MMP2, by sponging miR-214-3p	Upregulation	HLE B-3	Promoting	Yang et al. 2020)
		Induce KLF7, by sponging miR-1301-3p	Upregulation	ARPE-19	Promoting	Guo et al. 2022)
	lncRNA MIAT	Induce SP1, by sponging miR-29b	Upregulation	rMC-1	Promoting	Zhang et al. 2017)
	lncRNA NEAT1	Induce BDNF, by sponging miR-497	Downregulation	Rat	Suppressing	Li 2018)
		Induce YY1, by sponging miR-205	Downregulation	human	Suppressing	Li et al. 2020)
	lncRNA LUADT1	Induce PRX3, by sponging miR-383	Downregulation	RPEpiC, h1RPE7	Suppressing	Dai et al. 2022)
	lncRNA XIST	Induce SMAD2, by sponging miR-34a	Upregulation	SRA01/04	Suppressing	Wang et al. 2022a)
	lncRNA AK077216	Sponge miR-383	Downregulation	ARPE-19	Suppressing	Zhang et al. 2019)
	lncRNA SNHG16	Induce E2F1, by sponging miR-20a-5p	Upregulation	human	Promoting	Li et al. 2021c)
	lncRNA RPSAP52	Induce Timp3, by sponging miR-365	Downregulation	human	Suppressing	Niu et al. 2020)
	lncRNA FLG-AS1	Induce SOCS6, by sponging miR-380-3p	Downregulation	ARPE-19	Suppressing	Luo et al. 2022)
	lncRNA SNHG4	Induce Oxr1, by sponging miR-200b	Downregulation	ARPE-19	Suppressing	Yu et al. 2023)
	lncRNA MALAT1	Induce PDE6G, by sponging miR-378a-3p	Upregulation	RMECs	Suppressing	Li 2024)
	lncRNA MCM3AP-AS1	Induce SIRT1, by sponging miR-211	Downregulation	ARPE-19	Suppressing	Xia et al. 2022b)
CircRNA	CircZNF532	Induce STAT3, by sponging miR-20b-5p	Upregulation	ARPE-19	Promoting	Liang et al. 2022)
	CircPAG1	Induce E2F3, by sponging miR-211-5p	Downregulation	SRA01/04	Suppressing	Tao et al. 2022)
	CircFTO	Induce TGFA, by sponging miR-148a-3p	Upregulation	ARPE-19	Promoting	Huang et al. 2022)
	Circ_NNT	Induce TIMP3, by sponging miR-320b	Downregulation	ARPE-19	Suppressing	Liu et al. 2022)
	Circ_0000615	Induce YAP1, by sponging miR-646	Upregulation	HRPE	Promoting	Zeng et al. 2022)

## Major signaling molecules in retinal apoptosis

### PI3K/AKT/mTOR in Retinal cells apoptosis

The PI3K/AKT/mTOR signaling cascade constitutes a pivotal pathway engaged in fundamental biological processes, including angiogenesis, apoptosis, proliferation, growth, and cellular metabolism (Karar and Maity 2011; Yu and Cui 2016). The PI3K/AKT signaling pathway entails a series of events, primarily involving the binding of extracellular factors to receptors, subsequent receptor activation leading to the phosphorylation of PI3K, PI3K-mediated phosphorylation of AKT, and the subsequent activation of downstream effector molecules. Following activation, PI3K facilitates PIP2 phosphorylation at the 3-position of the inositol ring, resulting in the production of PIP3 (Weernink et al. 2004). Subsequently, PIP3 functions as a docking site for two protein kinases, specifically AKT, also known as PDK1 (phosphoinositide-dependent protein kinase 1), and protein kinase B (PKB), which are recruited to the cellular membrane through their pleckstrin homology interaction domains (PH domains) (Cho and Park 2008; Powis et al. 2023). Upon being recruited to the cellular membrane, AKT is phosphorylated at Ser473 by mTORC2 (mTOR complex 2), which induces a structural alteration in AKT and facilitates its subsequent phosphorylation at Thr308 by PDK1 (Moore et al. 2011; Pullen et al. 1998). Upon being activated, AKT experiences a series of phosphorylation events on its target proteins. These initial phosphorylations take place at the cellular membrane, enabling the subsequent detachment of AKT. Subsequently, AKT relocates to the cytoplasm and nucleus, where it proceeds to phosphorylate further target proteins. Target proteins phosphorylation culminates in the activation of growth, cell survival, and proliferation (Chen et al. 2022). PI3K/AKT signaling pathway a critical influence on apoptosis within retinal cells, thereby potentially contributing to the pathogenesis of DR.. In this regard, Zeng et al. documented a concurrent decline in DJ-1 protein levels and a rise in the apoptotic activity of RRPs within the HG group. Their further investigation revealed that exposure to HG concentrations for a duration of two days led to noteworthy levels of apoptosis in RRPs. This cellular condition demonstrated increased levels of ROS, along with an increase in p-p53 and activation of caspase-3. Furthermore, there was evidence of mitochondrial impairment, coupled with reduced catalase (CAT) and manganese superoxide dismutase (MnSOD) activities. Furthermore, a decline in DJ-1 protein expression, along with diminished levels of its subsequent targets, phosphorylation state of AKT, and mTOR was evident. Conversely, elevation of DJ-1/PARK7 led to contrasting outcome. Additionally, it enhanced activities of MnSOD and CAT, thus improving mitochondrial functionality. Furthermore, the increased expression of DJ-1/PARK7 was linked to a decrease in the expression of genes involved in apoptosis, specifically p-p53 and activated caspase-3. This led to a decline in ROS production and a lower rate of apoptosis in renal proximal tubular cells exposed to HG levels. These findings posit a protective role for DJ-1 in shielding retinal pericytes from oxidative stress damage induced by HG exposure. Consequently, DJ-1 has the potential to augment mitochondrial function, mitigate ROS generation, and bolster antioxidant capacity, thereby mitigating apoptosis in retinal pericytes. This effect might be facilitated via the PI3K/AKT/mTOR signaling pathway, which possibility associated with the initial pathophysiological processes DR (Zeng et al. 2019).

### TLR4/NF-κB in Retinal cells apoptosis

The TLR4/NF-κB pathway is a pivotal inflammatory signaling cascade intricately associated with cellular apoptosis, proliferation, differentiation, and the activation of pro-inflammatory responses. Toll-like receptors (TLRs) constitute a category of receptors that are classified within transmembrane proteins known as pattern recognition receptors (PRRs) (Kawai and Akira 2010; Zhu et al. 2018a). Among mammals, PRRs are unique molecules capable of transmitting extracellular antigenic signals to cells, thereby initiating an inflammatory response. TLR4 holds the distinction of being the inaugural member identified within the Toll-like receptor family, and it functions in both immune regulation and immune defense (Zhang et al. 2022). When activated, TLR4 undergoes dimerization and initiates two primary signaling pathways: one dependent on myeloid differential factor 88 (MyD88) and the other on toll/interleukin 1 receptor domain-containing adaptor inducing interferon-beta (TRIF). These signaling cascades subsequently lead to the activation of downstream factors such as mitogen-activated protein kinases (MAPKs), and NF-κB, ultimately inducing a diverse array of pro-inflammatory genes, including cytokines and enzymes associated with inflammation (Yu et al. 2022; Fitzgerald et al. 2001; Piras and Selvarajoo 2014). Recent experimental findings suggests that the TLR4/NF-κB signaling pathway assumes a pivotal role in the regulation of apoptosis in retinal cells and contributes significantly to the progression of DR. In this context, according to recent experimental findings, HG elevated the expression levels of TLR4, and four downstream signaling molecules associated with TLR4 (NLRP3, TRAF6, NF-κB, and MyD88) and pro-inflammatory cytokines (IL-18 and IL-1β) in retinal ganglion cells (RGCs). Notably, HG trigger a conspicuous elevation in the apoptosis of RGCs. Furthermore, TAK-242 introduction, which functions as an antagonist of TLR4, effectively suppressed both inflammation and RGC apoptosis within the HG group. These findings unequivocally illustrated the pivotal involvement of TLR4 in the inflammatory response and apoptotic processes of RGCs triggered by HG (Hu et al. 2017). Furthermore, Zhai et al. revealed that Berberine (BBR) led to a reduction in the ganglion cell layer, mitigated cellular apoptosis, attenuated oxidative stress induced by diabetes, and suppressed the NF-κB signaling pathway in a DR rat model. They subsequently unveiled that HG heightened oxidative stress and prompted mitochondria-dependent apoptosis in Müller cells by activating the NF-κB signaling pathway. As well, BBR reversed the influences induced by HG by reducing IκB phosphorylation, restraining NF-κB nuclear translocation, and consequently suppressing the NF-κB signaling pathway. In this manner, BBR conferred protection against DR by suppressing oxidative stress and cellular apoptosis through NF-κB signaling pathway deactivation (Zhai et al. 2020).

### SIRT1 in Retinal cells apoptosis

**Fig. 2:**
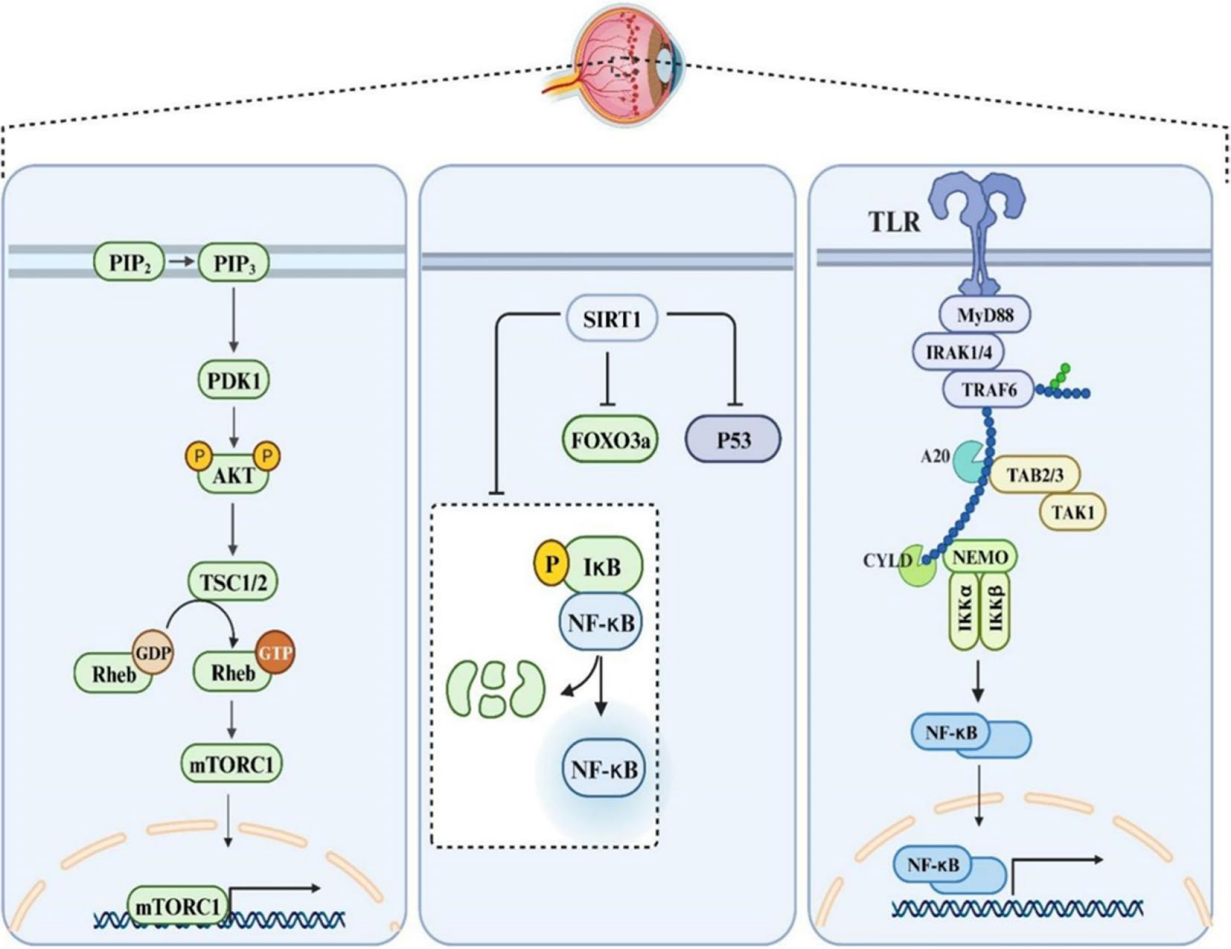
Integrative representation elucidating the pivotal signaling pathways orchestrating retinal cell apoptosis in the progression of DR. The diagram highlights key pathways, including SIRT1, TLR4/NF-κB, and PI3K/AKT/mTOR, and their intricate interplay in the regulation of apoptotic events within retinal cells

Sirtuins constitute a group of NAD^+^-dependent deacetylases that have maintained a high degree of conservation throughout the course of evolution. The sirtuin family, consisting of seven members, namely SIRT1-SIRT7, exhibits ubiquitous cellular distribution and is characterized by unique enzymatic properties, subcellular localization patterns, and physiological roles (Winnik et al. 2015). SIRT1 engages with protein substrates across diverse signaling pathways, including Wnt and Notch, and assumes a pivotal regulatory position in the control of numerous physiological functions within the body, prominently influencing processes such as metabolism, apoptosis, differentiation, and cell proliferation, thereby garnering significant interest from researchers across various fields of study. Recent experimental findings have revealed that agents promoting SIRT1 activation lead to reduced levels of apoptosis, inflammation, oxidative stress, and mitochondrial impairment, thereby conferring protection against DR (Karbasforooshan and Karimi 2018). In this regard, it was observed that lentiviral overexpression vector pLV5-Sirt increased cellular viability, and reduced apoptotic rate in RGCs, while pLV3-si-Sirt1 exert opposite effects. Also, pLV5-Sirt1 significantly reduce the expression levels of FOXO3a, p53, caspase-3, and NF-κB within RGCs, while pLV3-si-Sirt1 exert opposite effects. In this manner, Sirt1 has the capacity to suppress RGC apoptosis by modulating the expression of certain apoptotic cytokines, making it a potential candidate gene for therapeutic interventions in DR (Zhou et al. 2021) (Fig. 2).

### Apoptosis-related miRNA in DR

miRNAs constitute a class of short, ncRNA molecules, typically ranging from 19 to 25 nucleotides in length, that function as post-transcriptional regulators of gene expression via silencing mechanisms (Lu and Rothenberg 2018). Their biogenesis entails a two-step cleavage process wherein a progressively shorter hairpin structure is generated: the initial cleavage orchestrated by ribonuclease Drosha and Dgcr8, followed by a subsequent cleavage event led by Dicer alone, culminating in the formation of a miRNA duplex. The miRNA duplex is loaded onto an Argonaute protein, forming the basis of the miRNA-induced silencing complex (miRISC), designated as the passenger strand, undergoes extrusion. The miRISC associates with 3'-UTR of target mRNA sequences, subsequently initiating either mRNA degradation or translational inhibition (Cai et al. 2009; Liu et al. 2008). Recent experimental evidence has demonstrated that miRNAs significantly contribute to the development of DR by predominantly regulating apoptotic pathways. In this regard, the downregulation of miR-122 alleviated apoptosis triggered by HG in ARPE-19 cells. Additionally, HG resulted in downregulation of Bcl-2 and upregulation of cleaved caspase-3 in the cells. Interestingly, these effects were abrogated by a subsequent reduction in miR-122 expression. Furthermore, miR-122 predominantly functions by targeting Tissue inhibitor of metalloproteinases-3 (TIMP3). Also, simultaneous increase in TIMP3 expression counteracted the effect of elevated miR-122 levels on HG-induced apoptosis in ARPE-19 cells. In conclusion, miR-122 promoted apoptosis in ARPE-19 cells and accelerated the advancement of DR by suppressing TIMP3 (Wang et al. 2020a). In the subsequent section, we will discuss relevant literature concerning miRNAs function in apoptotic processes in DR.

#### MiR-200a

The miR-200 family comprises five members (miR-429, -141, and 200ca/b/c) which are arranged in two separate polycistronic primary miRNA transcripts, specifically miR-200c-141, and miR-200b-200a-429, located on human chromosomes 12 and 1, respectively (Fu et al. 2019). ARPE-19 cells subjected to HG demonstrated a notable decrease in the expression of miR-200a-3p, along with a simultaneous increase in transforming growth factor-β2 (TGF-β2) levels. This finding was similarly observed in the retinal tissues of rats with DR. ARPE-19 cells exposed to HG exhibited a significantly increased incidence of apoptosis relative to the control. Moreover, HG induced a marked upregulation of pro-apoptotic proteins, including Bax and Caspase-3. Conversely, a concomitant downregulation of the anti-apoptotic protein Bcl-2 was observed. Moreover, upregulation of miR-200a-3p resulted in a significant decrease in the apoptotic rate of ARPE-19 cells exposed to HG conditions. Mechanistically, increased miR-200a-3p levels were observed to attenuate apoptosis in ARPE-19 cells under HG conditions, mediated by the inhibition of the TGF-β2/Smad signaling pathway. In conclusion, increased expression of miR-200a-3p effectively suppress apoptosis in DR by blocking the TGF-β2/Smad pathway, indicating a promising therapeutic marker for the treatment of DR (Xue et al. 2020). Additionally, miR-200b display a substantial upregulation in the retinas of Akita mice. miR-200b directly regulates the expression of Oxidation resistance 1 (Oxr1). This regulatory interaction was demonstrated in a human Müller cell line (MIO-M1), where overexpression of miR-200b resulted in a decrease in Oxr1 expression levels. Also, upregulation of recombinant Oxr1 mitigated oxidative stress markers, specifically the nitration of cellular proteins, and also alleviated apoptosis triggered by 4-hydroxynonenal (4-HNE), which is an oxidative stress-inducing agent. Furthermore, miR-200b inhibitor reduced the number of apoptotic cells, while transfection with a miR-200b mimic increased the apoptotic cell count after exposure to 4-HNE treatment. Thereby, miR-200b, through the regulation of Oxr1 and its modulation of apoptosis, potentially plays a protective role in DR (Murray et al. 2013)..

#### MiR-423-5p

MiR-423 is situated on chromosome 17 at the 17q11.2 locus (gene coding ID: 494335). Two mature miRNA sequences, specifically miR-423-5p and miR-423-3p, were discerned through an exploration of the miRBase sequence database (Ke et al. 2020). MiR-423-5p expression demonstrates a significant increase in RPE cells exposed to HG conditions, as well as in the plasma of DR patients. Furthermore, the upregulation of miR-423-5p worsens apoptosis induced by HG. Functionality, miR-423-5p directly targets TFF1, leading to the suppression of TFF1 expression, and that upregulating TFF1 mitigates apoptosis in RPE cells subjected to HG. Moreover, increased expression of TFF1 negates the pro-apoptotic effects induced by miR-423-5p in RPE cells exposed to HG. Hence, MiR-423-5p promotes apoptosis in RPE cells under HG conditions by inhibiting TFF1. Furthermore, reduction of TFF1 led to the activation of the NF-κB pathway and enhanced apoptosis induced by HG in RPE cells. Thereby, miR-423-5p modulate apoptosis induced by HG in RPE cells by suppressing the NF-κB signaling pathway mediated through TFF1. Importantly, NFE2, through miR-423-5p overexpression in RPE cells subjected to HG conditions, modulates the NF-κB signaling pathway mediated by TFF1. Therefore, a regulatory mechanism comprising NFE2, miR-423-5p, and NF-κB emerges as a significant determinant affecting apoptosis in RPE cells induced by HG (Xiao et al. 2019).

#### MiR-455-5p

Human miR-455 resides within the 9q32 locus on chromosome 9. Interestingly, the COL27A1 gene, encoding the collagen type XXVII alpha 1 chain, harbors the miR-455 sequence (Kumar and Reddy 2019). The involvement of miR-455 has been suggested in a range of human conditions, including DM. miR-455-5p markedly reduce in ARPE-19 cells subjected to HG. As well, enforced expression of miR-455-5p resulted in changes to the Bax/Bcl-2 ratio and cleaved caspase-3, leading to a notable increase in cellular viability and a reduction in apoptosis in HG conditions. Furthermore, elevation of miR-455-5p functioned as a negative regulator of intracellular ROS levels, decreased malondialdehyde (MDA) content, and decreased NADPH oxidase 4 expression, while enhanced the activities of GPX, catalase, and superoxide dismutase under HG conditions. So, miR-455-5p significantly attenuated oxidative stress injury induced by HG. Most importantly, miR-455-5p directly target suppressor of cytokine signaling 3 (SOCS3), and miR-455-5p exerts a negative regulatory influence on the expression of SOCS3. Moreover, SOCS3 restoration nullified the advantageous impacts of miR-455-5p on apoptosis and the buildup of ROS. Taken together, miR-455-5p mitigated HG-induced damage by inhibiting apoptosis and oxidative stress through the targeting of SOCS3, suggesting that miR-455-5p emerges as a promising candidate for future DR therapeutic development (Chen et al. 2019).

#### MiR-29a/b

Within the human genome, the miR-29 family comprises four members: miR-29b-1, miR-29b-2, miR-29a, and miR-29c. These miRNAs originate from two distinct primary transcripts: pri-miR-29a/b1 cluster located on chromosome 7q32.3 and pri-miR-29b2/c cluster situated on chromosome 1q32.2 (Horita et al. 2021). The miR-29 family is frequently associated with DM in various studies. In diabetic patients, miR-29a expression shows downregulation, while STAT3 and IL-6 levels exhibit upregulation in both the lens capsules and aqueous humor. As well, Overexpression of miR-29a combined with si-STAT3 transfection mitigated the increase in apoptosis and the disruption of MMP caused by HG. Notably, suppression of STAT3 resulted in an elevated Bcl-2/Bax ratio when compared to cells subjected to HG treatment alone. As well, it was observed that STAT3 upregulation countered the impacts of miR-29a transfection. Furthermore, by suppressing STAT3 signaling pathway, miR-29a alleviates the adverse impacts of HG on cell viability and mitochondrial dysfunction. Also, upregulation of miR-29a led to a partial decrease in ROS generation, elevated MDA levels, reduced SOD activity, and an upregulation of the Bcl-2/Bax ratio, which are linked to apoptotic pathways. These findings underscore miR-29a involvement in oxidative damage and apoptotic processes. In this manner, HG induces inflammatory responses and initiates mitochondrial dysfunction, thereby augmenting apoptosis via activation of the IL-6/STAT3 pathway (Li et al. 2021b). On the other hand, numerous studies have consistently shown that miR-29 functions as a proapoptotic factor in this particular condition. In this context, HG resulted in a concentration-dependent increase in apoptotic cell death within RPE cells, and miR-29 exhibited an upregulation in response to HG within RPE cells. Additionally, Downregulation of miR-29 expression mitigated HG-induced apoptosis in RPE cells, as evidenced by a reduction in caspase-7 protein generation. Also, miR-29 directly target PTEN, and downregulation of miR-29 increased PTEN expression. Thereby, miR-29 contributes to the apoptotic pathway triggered by HG in RPE cells. This effect is likely mediated by its inverse association with PTEN expression (Lin et al. 2016). Furthermore, Zeng et al. discovered that the miR-29b-3p expression exhibited a 3.2-fold increase, while the SIRT1 protein was reduced in patients with DR. In the HG-CoCl2 environment, they observed an elevation in miR-29b-3p and the Bax/Bcl-2 ratio, accompanied by a reduction in SIRT1 within HRMECs. They observed a decrease in the viability of HRMECs and a concomitant rise in apoptotic cell death when exposed to HG-CoCl2 treatment. Ultimately, they demonstrated that miR-29b directly target SIRT1, and that the Up-regulation of miR-29b-3p corresponded with a significant down-regulation of SIRT1 protein expression and a concomitant increase in the Bax/Bcl-2 ratio. Conversely, down-regulation of miR-29b-3p resulted in opposite effects. Furthermore, SRT1720, a SIRT1 agonist, mitigated the apoptosis of HRMECs induced by miR-29b-3p by enhancing the expression of SIRT1 protein. So, aberrant regulation of miR-29b-3p/SIRT1 axis is a crucial mechanism driving the apoptosis of HRMECs in DR (Zeng et al. 2020).

#### MiR-204

The miR-204 resides within the sixth intron of the transient receptor potential melastatin 3 (TRPM3) gene.. MiR-204 assumes a pivotal role in both the developmental processes and functional aspects of the retina, with its dysregulation being implicated in the pathogenesis of various retinopathies (Bereimipour et al. 2021). Quantitative analysis of miR-204 expression in the retinal tissues of DR model rats revealed a significant downregulation in comparison to healthy controls. This observation suggests a dysregulation of miR-204 in DR pathogenesis, potentially contributing to the development of the disease. Moreover, Transfection with miR-204 mimics resulted in a marked upregulation of endogenous miR-204 expression. This, in turn, mediated a coordinated increase in the expression of Bcl-2 and SIRT1, while concurrently suppressing the production of pro-inflammatory mediators like TNF-α. Thus, miR-204 mitigates inflammation and cellular apoptosis in DR in rats via an increase in Bcl-2 and SIRT1 expression (Qi et al. 2020).

#### MiR-211

MiR-211 is produced through the transcriptional process of Trpm1 gene. It is important to note that Trpm1 gene is located on chromosome 15q13-q14, a genomic region commonly associated with deletions found in various cancerous conditions. Furthermore, MiR-211 is situated specifically within the sixth intron of the Trpm1 gene (Yuan et al. 2022; Ye et al. 2022). MiR-211 may play a pivotal role in regulating the metabolic and catabolic processes of retinal cells, with its importance extending to the maintenance of adult visual functionality (Barbato et al. 2017). Diabetic cataract (DC) mice exhibited elevated levels of p53, Bax, and miR-211, alongside reduced levels of SIRT1 and Bcl-2. As well, miR-211 functions as a direct regulator of SIRT1 expression. Furthermore, upregulation of miR-211 trigger elevated expressions of p53, Bax, and miR-211 alongside reduced expressions of SIRT1 and Bcl-2. Notably, elevated expression of miR-211 led to decreased proliferation, and increased apoptosis of lens epithelial cells, while miR-211 depletion is associated with opposite effects. Therefore, miR-211 may function as a regulator of lens epithelial cell homeostasis in DC mice. miR-211 overexpression appears to promote apoptosis and inhibit proliferation, potentially through its interaction with SIRT1. These findings implies a potential therapeutic role for miR-211 in the treatment of DR (Fitzgerald et al. 2001).

#### MiR-365

Zheng et al. explored miR-365 influence on retinal neuron apoptosis in rats with diabetes mellitus, primarily through its targeting of IGF-1. In their research, suppression of miR-365 through the administration of anti-miR-365 led to a reduction in apoptotic cells and Bax protein levels relative to control groups. Conversely, the groups treated with sh-IGF-1 and anti-miR-365 + sh-IGF-1 exhibited a contrasting pattern of results. Furthermore, the diabetes mellitus rat models displayed elevated levels of miR-365 and Bax expression, alongside reduced levels of Bcl-2 and IGF-1 expression. Additionally, in the diabetic rat models, a higher quantity of cells undergoing apoptosis was noted. Furthermore, they demonstrated that the sh-IGF-1 group exhibited decreased Bax expression, increased Bcl-2 and IGF-1 expressions, and a reduced number of apoptotic cells. Lastly, they observed that the anti-miR-365 + sh-IGF-1 groups displayed an upregulation of Bax expression, downregulation of IGF-1 and Bcl-2 expressions, and an increased count of apoptotic cells when compared to the anti-miR-365 group. Their findings indicate that the inhibition of miR-365 results in heightened expression of IGF-1, which subsequently exerts anti-apoptotic effects on retinal neurons in diabetic rats. These results underscore the potential of miR-365 as a viable therapeutic target in the context of DR (Zheng et al. 2018).

#### MiR-126

MiR-126, predominantly known as miR-126-3p,is situated within intron 7 of the EGFL7 gene on chromosome 9 of the human genome (Nikolic et al. 2010). Dysregulation of miR-126 constitutes a key element in the etiology and development of DM and its associated complications. IL-17A increased, while miR-126 displayed upregulation in HRECs exposed to HG. Functionality, miR-126 upregulation stimulated cell proliferation and hindered apoptosis process in HRECs subjected to HG conditions. Interestingly, introduction of IL-17A abrogated the effects mediated by miR-126. Additionally, miR-126 exerted suppressive effects on the expression of caspase-3, Bax, and IL-17A, concurrently promoting survivin expression and inducing phosphorylation of both AKT and PI3K. Importantly, the reintroduction of IL-17A mitigated these effects. Notably, IL-17A function as a direct target of miR-126, and overexpression of miR-126 decreased IL-17A. In this manner, miR-126 enhances cell growth and prevents cellular apoptosis in HRECs exposed to HG by stimulating the PI3K-AKT pathway, elevating survivin levels, and reducing the levels of caspase-3 and Bax through the regulation of IL-17A (Chen et al. 2020a).

#### MiR-21

The mature form of miR-21 displays complete conservation among mammals, similar to numerous other microRNAs, and is generated from a single gene. The human miR-21 gene has been thoroughly characterized and is situated on chromosome 17q23.2 (Olivieri et al. 2021). Recent investigations have supplied supporting evidence that the expression levels of plasma miR-21 can serve as a reliable marker to gauge the severity of T2D accompanied by DR (Jiang et al. 2017). Chen et al. via experimental investigation revealed a decreased level of retinal cell apoptosis in dKO mice when contrasted with control db/db mice. This observation implies that the deletion of miR-21 led to a reduction in diabetes-induced retinal cell apoptosis. They also revealed an upregulation of PPARα in miR-21 knockout (miR-21^−/−^) OIR retinas when compared to wild-type (WT) OIR retinas, thereby confirming the involvement of miR-21 in the downregulation of PPARα in retinal tissue subjected to ischemic conditions. Furthermore, a notable reduction in retinal cell apoptosis, as evidenced by reduced DNA fragmentation, was observed in miR-21^−/−^ OIR retinas in comparison to WT OIR retinas. These results indicate that the miR-21 silencing mitigated the reduction of PPARα induced by ischemia and improved the condition of retinal apoptosis. In summary, downregulation of PPARα and the induction of apoptosis in DR are, to some extent, mediated by the heightened expression of miR-21 within the diabetic retina (Chen et al. 2017a).

#### MiR-146a

The miR-146a gene is situated on human chromosomes 19, 10, and 5, whereas in mice, it is located on chromosome 11. A substantial body of medical research has consistently identified dysregulation of miR-146a in individuals with T2D, underscoring the significant role of miR-146a in the development of T2D and its associated complications (Ghaffari et al. 2023). In human RECs exposed to HG conditions, upregulation of miR-146a expression led to diminished levels of IL-6, reduced STAT3 phosphorylation, and decreased VEGF. Additionally, it resulted in attenuated cellular apoptosis. More importantly, in RECs, miR-146a modulates STAT3/VEGF signaling and apoptosis via the IL-6 receptor signaling pathway. Importantly, miR-146a effectively inhibited IL-6 signaling, consequently resulting in diminished levels of VEGF and STAT3 in RECs under HG conditions, ultimately leading to a reduction in apoptosis. Thereby, miR-146a emerges as a potential therapeutic target in DR due to its capacity to suppress the IL-6-mediated STAT3/VEGF pathway, thereby mitigating both apoptosis and inflammation (Ye and Steinle 2017).

#### MiR-219-5p

HG treatment induced a significant upregulation of miR-219-5p expression. Also, miR-219-5p was found to directly target the liver receptor homolog-1 (LRH-1), resulting in a significant decrease in LRH-1 levels in ARPE-19 cells under HG conditions. As well, inhibition of miR-219-5p significantly attenuated HG-induced apoptosis in ARPE-19 cells, accompanied by a concomitant increase in cell viability. Furthermore, miR-219-5p silencing activate LRH-1/Wnt/β-Catenin signaling pathway. In conclusion, involvement of miR-219-5p in DR pathogenesis is attributed to its impact on the apoptosis of human RPE cells via LRH-1/Wnt/β-Catenin signaling pathway (Zhao et al. 2018).

#### MiR-495

MiR-495 represents a small non-coding RNA entity originating from a genetic locus situated on chromosome 14, specifically at the chromosomal region 14q32.31 (Chen et al. 2017b). Existing finding has suggested that miR-495-3p plays a crucial function in cancer progression (Liang et al. 2015). miR-495 substantially increased in RGCs subjected to HG treatment. MiR-495 reduction provided protection to RGCs against HG-induced apoptosis, while conversely, the miR-495 upregulation yielded the opposite effect. Notably, miR-495 serves as a direct regulator of Notch1, as miR-495 exhibited a negative regulatory effect on Notch1 expression and the associated Notch signaling pathway. Moreover, miR-495 reduction resulted in the suppression of PTEN expression and the activation of Akt. Importantly, the inhibition of miR-495 provides a protective effect against HG-induced apoptosis; however, this effect is effectively nullified upon Notch1 silencing. Overall, miR-495 reduction mitigates apoptosis in RGCs induced by HG through the regulation of the Notch1-mediated PTEN/Akt signaling pathway (Zhang et al. 2018).

#### MiR-383

miR-383 consists of two unique mature variants, namely miR-383-3p and miR-383-5p. The miR-383 gene is situated on chromosome 8p22, specifically positioned within the third intron of the sarcoglycan zeta (SGCZ) gene (Yi et al. 2022). miR-383 levels increase significantly in ARPE-19 human RPE cell lines in response to HG exposure. Additionally, overexpression of miR-383 by altering the expression of Bcl-2 and Bax led to diminished cell viability, enhanced apoptosis, and increased ROS production in ARPE-19 cells. Importantly, miR-383 directly target peroxiredoxin 3 (PRDX3), and downregulated its expression in ARPE-19 cells. Furthermore, reintroduction of PRDX3 counteracted the miR-383-induced increase in ROS production and apoptosis. Conversely, the suppression of PRDX3 mimicked the miR-383 deleterious effects on ARPE-19 cells. In summary, HG-induced upregulation of miR-383 mechanistically contributes to apoptosis in RPE cells, likely by repressing PRDX3 expression (Jiang et al. 2017).

#### MiR-218

MiR-218 is a type of intronic miRNA that is uniquely present in vertebrates. This gene exhibits co-expression with its host genes, the tumor suppressor genes SLIT2 and SLIT3. miR-218 arises from two discrete genomic loci: miR-218–1 and miR-218–2. These genetic loci have been assigned to chromosomal regions 4p15.31 and 5q35.1 (Lu et al. 2015). Elevated glucose levels caused a rise in miR-218 expression, which subsequently inhibited cell proliferation and triggered apoptosis in ARPE-19 cells. Furthermore, miR-218 overexpression inhibited ARPE-19 cell proliferation and promoted apoptosis, while miR-218 depletion led to contrary outcomes. Mechanistically, Runx2 acted as a target of miR-218 to assist in restraining cell proliferation and promoting apoptosis in ARPE-19 cells. Therefore, miR-218 promotes apoptosis in RPEs through direct targeting of RUNX2, suggesting the miR-218/RUNX2 regulatory axis as a promising target for DR (Yao et al. 2019).

#### MiR-542-5p

MiR-542-5p, situated in a genomic region on chromosome Xq, possesses the capability to suppress hyperlipidemia and hyperglycemia (Tian et al. 2020). MiR-542-5p display a downregulation in individuals with DR and in retinal pigment epithelial (RPE) cells treated with HG. Upregulation of miR-542-5p effectively curbed apoptosis in RPE cells subjected to HG conditions. Furthermore, miR-542-5p was found to directly interact with co-activator-associated arginine methyltransferase 1 (CARM1). Overexpression of miR-542-5p caused a decrease in CARM1 expression levels, whereas suppression of miR-542-5p led to an elevation in CARM1 levels. Importantly, upregulation of CARM1 promoted apoptosis mediated by miR-542-5p in RPE cells exposed to HG conditions. Thus, miR-542-5p's direct targeting of CARM1 suggests a protective role for miR-542-5p in RPE cells, opening new avenues for therapeutic interventions in DR (Guo et al. 2020).

#### MiR-133b

The miR-206/miR-133b cluster is remarkably preserved in the muscle tissues of various organisms, including flies, mice, and humans, and it is situated on chromosome 6p12.2 (Nohata et al. 2012). Yao et al. noted that texposure to HG or miR-133b silencing result in a decrease in apoptosis in hRECs. Furthermore, HG resulted in an increase in the mRNA and protein levels of LIMK, ROCK1, RhoA, and the p-MLC protein in hRECs. On contrary, elevated expression of miR-133b suppressed cellular proliferation, enhanced apoptosis, and led to a downregulation of both mRNA and protein quantities of RhoA, ROCK1, LIMK, and p-MLC in hRECs subjected to HG conditions. Thereby, heightened expression of miR-133b hindered cell proliferation and stimulated apoptosis in a DR cell model through the reduction of RhoA expression (Yao et al. 2018).

#### MiR-499-3p

Liu et al. demonstrated a decrease in IFNA2 levels and an increase in miR-499-3p expression in retinal tissues and cells of diabetic rats. This molecular alteration resulted in the activation of the TLR4 signaling pathway. They ascertained that miR-499-3p function as a direct regulator of IFNA2, and exerting an inhibitory effect on its expression. Also, miR-499-3p may function as a potential regulator of the TLR4 signaling pathway by targeting IFNA2. Their further experiments revealed that the reduction in miR-499-3p levels facilitated the proliferation of retinal cells and concurrently inhibited apoptosis, thereby mitigating the effects of DR. In conclusion, miR-499-3p facilitated the progression of retinopathy by activating TLR4 signaling pathway and promoting apoptosis, underscoring its potential for exploration as a therapeutic target for further research in DR (Liu et al. 2020).

#### MiR-34a

Residing on chromosome 1p36, miR-34a) has been implicated in the pathophysiology of DM (Mone et al. 2022). MiR-34a upregulated, while SIRT1 downregulated in retinal tissues of DR-afflicted rats and in HG-induced RVECs. Suppression of miR-34a had a beneficial impact on DR, exerting its influence by modulating apoptosis and the expression of VEGF in DR rats. As well, miR-34a directly target SIRT1, and overexpression of miR-34a suppressed proliferation and induced apoptosis in RVECs, and these effects successfully reversed by increasing SIRT1 expression. Therefore, miR-34a drives apoptosis in RVECs by modulating SIRT1 in DR rats, underscoring the potential significance of the miR-34a/SIRT1 axis as a potential avenue for DR treatment (Ji et al. 2020). Furthermore, Ma et al. noted that retinal cell apoptosis, the expression levels of miR-34α, NF-κB, HMGB1, and caspase-3 increased in rats with DR, while miR-34α silencing exert opposite effects.. In this manner, suppression of miR-34α mitigates retinal cell apoptosis in rats with DR by modulating the expression of HMGB1 and the downstream NF-κB pathway (Ma et al. 2020).

#### MiR-19a

Located within the third intron of the C13orf25 gene on human chromosome 13q31.3, the miR-17–92 cluster is a highly conserved gene cluster transcribed by a single polycistronic promoter (Ardizzone et al. 2021). Zhang et al. observed a marked reduction in miR-19a expression in DR rats treated with a miR-19a inhibitor. In their study, RGCs exhibited a well-organized arrangement, with indications of apoptosis and less severe necrosis observed in the miR-19a inhibitor group. miR-19a depletion led to a significant decrease in the percentage of apoptotic cells This was accompanied by a marked downregulation of PTEN protein expression and a concomitant upregulation of Akt pathway activity, compared to the DR group. MiR-19a specifically binds to the PTEN protein to regulate the PI3K/Akt signaling pathway, subsequently influencing RGCs apoptosis, and advancement of DR (Zhang and Liu 2020).

#### MiR-203a

MiR-203a resides within the human chromosome locus 14q32. This chromosomal region exhibits high levels of variation and harbors roughly 12% of all identified microRNA genes (Liu et al. 2019a). Zhang et al. explored the miR-203a-3p function in CoCl2-induced apoptosis of RPEs. They demonstrated the expression of miR-203a-3p within the RPE of both control and diabetic rat models, and identified miR-203a as a direct modulator of SOCS3. They disclosed that the introduction of miR-203a-3p mimics led to an enhancement in CoCl2-induced apoptosis of RPEs. However, the apoptotic effects of miR-203a-3p were partially counteracted by the increased expression of SOCS3 or the administration of the JNK inhibitor SP600125. So, miR-203a-3p functions as a significant novel regulator of apoptosis induced by CoCl2 in RPE cells, operating by regulating the expression of SOCS3. In conclusion, deregulation of miR-203a-3p/SOCS3/JNK/c-Jun signaling pathway is hypothesized to be a critical contributor to RPE cell apoptosis in DR (Zhang et al. 2020).

#### MiR- 296-5p

MiR-296 is situated within the chromosomal region 20q13.32, exhibiting substantial sequence conservation across species and exerting significant roles in various biological processes, including apoptosis (Zhu et al. 2018b). In DR mice, retinal tissues exhibited a marked increase in G protein subunit alpha i2 (GNAI2) and HDAC3 expression levels, accompanied by a significant decrease in miR-296-5p expression. The researchers noted that restoration of miR-296-5p or HDAC3 silencing resulted in a decrease in Evans blue leakage in DR mice. Additionally, it mitigated retinal ganglion cells apoptosis, decreased levels of MDA and VEGF, and increased SOD activity in both retinal tissues and serum of DR mice. Through interaction with the miR-296-5p promoter region, HDAC3 functioned as a suppressor of miR-296-5p expression, leading to a subsequent upregulation of GNAI2. In this manner, reducing HDAC3 levels or reinstating miR-296-5p mitigates the apoptosis of RGCs in DR mice by lowering the expression of GNAI2 (Che et al. 2022).

#### MiR-345-5p

MiR-345, a small nvRNA, is situated on human chromosome 14q32.2 and has been implicated in diverse human pathological conditions (Natesh et al. 2021). Ge et al. explored the Mbd2/miR-345-5p/Atf1 axis influence in the progression of DR. They noted that exposure to HG resulted in a significant upregulation of Mbd2, implicating its role in promoting apoptotic mechanisms within RGCs in HG-mediated apoptosis. Their investigation elucidated that Mbd2 contained putative binding sites for miR-345-5p, leading to a subsequent increase in miR-345-5p levels via enhanced promoter demethylation of Mbd2. Further investigations demonstrated that activating transcription factor 1 (Atf1) functioned as an anti-apoptotic factor within RGCsduring the apoptotic process, and was identified as a target gene of miR-345-5p. Moreover, they observed an elevated count of surviving RGCs in diabetic mice with Mbd2 knockout compared to wild-type mice, resulting in improved visual function. Collectively, in the retina, HG-induced upregulation of Mbd2 partially mediated retinal neuronal cell apoptosis by modulating miR-345-5p/Atf1 axis (Ge et al. 2021).

#### MiR-221

**Fig. 3:**
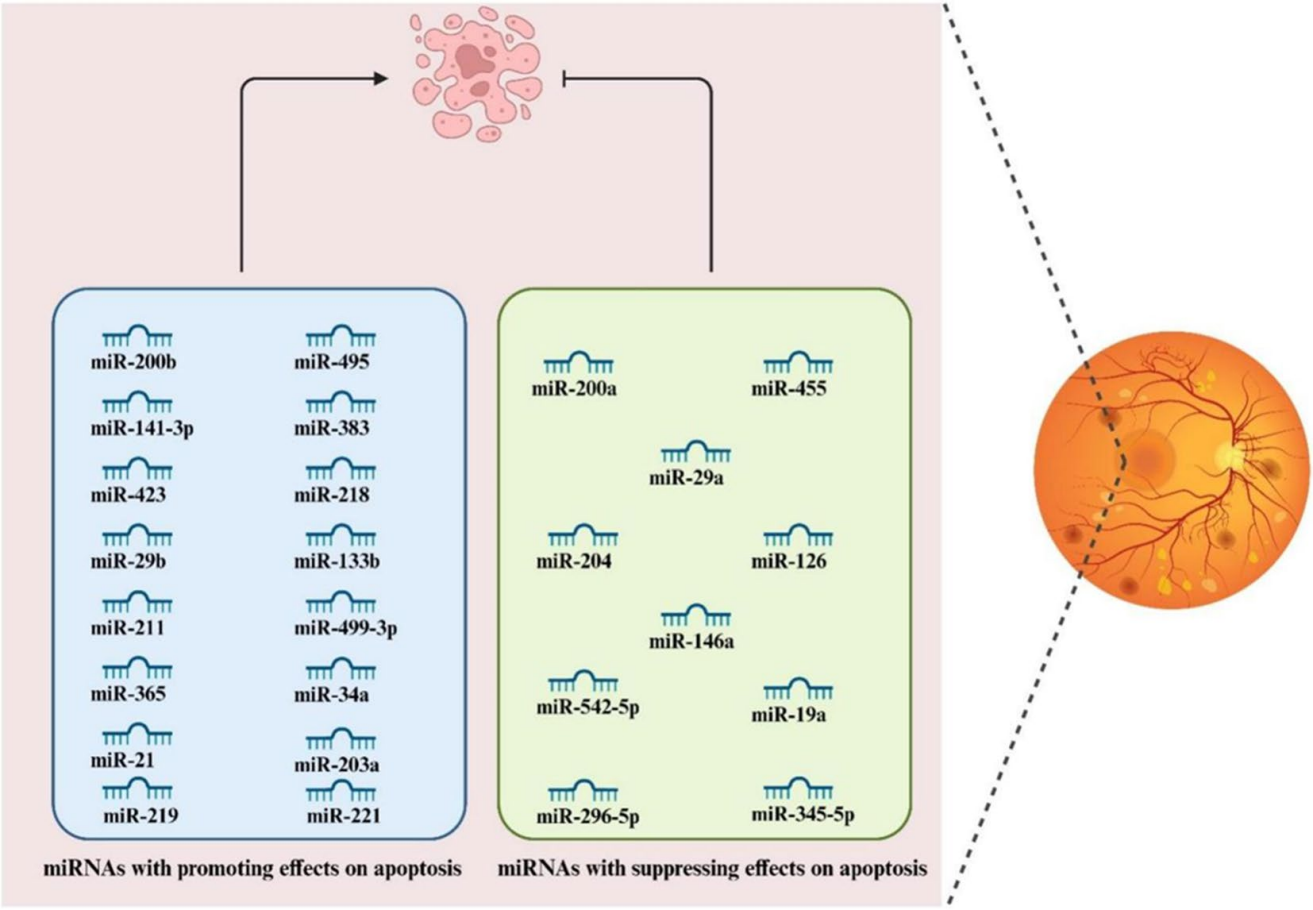
Illustration depicting the intricate network of apoptosis-related miRNAs implicated in the pathogenesis of DR. The visual narrative highlights key miRNAs and their regulatory roles in modulating apoptotic pathways within retinal cells, contributing to the progression of DR

Co-localized on the X chromosome at Xp11.3, miR-221 and miR-222 are highly conserved across vertebrates, sharing identical seed sequences and are separated by 727 bases (Li et al. 2023). Chen et al. observed that HG exposure induced an unregulation of miR-221 expression, while concurrently downregulating Nrf2 and SIRT1 levels. Importantly, the upregulation of miR-221 exacerbated apoptosis in the presence of HG conditions. Their in-depth analysis uncovered that miR-221 directly bind with SIRT1 transcript, thereby suppressing SIRT1 expression in hRMEC. This regulatory action of miR-221 subsequently hindered Nrf2 pathway and initiated apoptosis in hRMEC. In this manner, miR-221/SIRT1/Nrf2 signaling axis was identified as a promoter of apoptosis in hRMEC when exposed to HG conditions (Chen et al. 2020b) (Fig. 3).

## LncRNA/miRNA/mRNA axis-mediated apoptosis in DR

LncRNAs, which represent the largest subgroup of ncRNAs, are characterized as RNA molecules exceeding 200 bases in length. Their transcription is mediated by by RNA polymerase II and undergo capping at their 5' end and polyadenylation at their 3' end (Zhou et al. 2023). LncRNAs can be categorized into nuclear and cytoplasmic lncRNAs. Nuclear lncRNAs are primarily involved in the modulation of epigenetic processes and transcription, whereas cytoplasmic lncRNAs exert their influence at the translational level. LncRNAs harboring miRNA response elements (MREs) that are shared with coding RNAs, possess comparable miRNA target sequences, thus impeding the regulatory actions of miRNAs on mRNAs. So, these lncRNAs, recognized as competing endogenous RNAs (ceRNAs), function as 'miRNA sponges,' sequestering miRNAs and thereby promoting increased translation of their target mRNAs (Wang et al. 2020). Several investigations have provided evidence that the LncRNA/miRNA/mRNA axis can modulate the onset and advancement of DR by targeting specific mRNAs and controlling various cellular processes, including apoptosis of retinal cells. In the following section, we provide a thorough overview of the regulatory funcitons within the lncRNA–miRNA–mRNA network in retinal cells apoptosis and DR pathogenesis.

### LncRNA MEG3/miRNA axis

Xiao et al. reported silencing SOCS6 significantly reduced cell viability and abrogated the miR-19b depletion-induced increase in viability, suggesting direct targeting and downregulation of SOCS6 by miR-19b. In their study, miR-19b elevated cell apoptosis rates, enhanced caspase-3/7 activity, and heightened inflammatory factors by modulating the JAK2/STAT3 signaling pathway through SOCS6 mediation. Their subsequent experiments revealed that MEG3 mitigated HG-induced apoptosis in hRMECs by modulating the miR-19b/SOCS6 axis. Thereby, MEG3 effectively suppressed apoptosis and mitigated inflammation induced by HG through the modulation of the miR-19b/SOCS6 axis within the context of the JAK2/STAT3 signaling pathway in hRMECs. In this manner, lncRNA MEG3 / miR-19b / SOCS6 axis represents a potential avenue for addressing DR (Xiao et al. 2020). Similarly, Luo et al. revealed reduced expression levels of Nrf2, and lncRNA MEG3, along with elevated levels of miR-93, in both the blood samples obtained from DR patients and in human RPE cells, including those treated with HG, such as ARPE-19 cells. Their findings demonstrated that miR-93 overexpression led to a hindrance in cellular proliferation and an enhancement in apoptotic processes, whereas the upregulation of MEG3 or Nrf2 facilitated proliferation and concurrently suppressed apoptosis and inflammatory responses. In addition, MEG3 interacted with miR-93, resulting in the downregulation of miR-93 levels. Conversely, miR-93 directly targeted Nrf2, leading to its negative regulation. So, lncRNA MEG3 mitigates apoptosis and inflammation induced by HG in RPE cells by modulating miR-93/Nrf2 axis. This novel insight contributes to our understanding of the initiation and progression of DR (Luo et al. 2020). Additionally, Tong et al. investigated the signaling pathways underlying MEG3-mediated regulation of inflammation and apoptosis in DR. Within their study, HG exposure exhibited suppressive effects on SIRT1 and MEG3 expression, while concurrently inducing an upregulation of miR-34a levels. Their investigation unveiled that MEG3 could enhance the expression of SIRT1 through its interaction with miR-34a. Furthermore, HG-induced apoptosis and the release of inflammatory cytokines, including TNF-α, IL-6, and IL-1β, were attenuated by the upregulation of MEG3 and miR-34a silencing. However, the miR-34a upregulation counteracted these protective effects mediated by MEG3. So, overexpression of MEG3 resulted in the inhibition of the NF-κB signaling pathway and an elevation in the Bcl-2/Bax ratio, which was achieved through the downregulation of miR-34a. Thereby, MEG3 demonstrated the capability to mitigate apoptosis and inflammation induced by HG by suppressing the NF-κB signaling pathway via its interaction with miR-34a/SIRT1 axis. Therefore, MEG3/miR-34a/SIRT1 axis represents a promising target for therapeutic intervention in DR (Tong et al. 2019).

### LncRNA PVT1/miRNA axis

Experimental finding from Yang et al. unveiled an elevation in the expression of lncRNA PVT1 within the HLE B-3 cells induced by HG in contrast to the control group maintained under normal glucose conditions. Furthermore, they provided evidence indicating that the transcription factor SP1 exhibit potential DNA binding activity to PVT1 promoter region, thereby facilitating its transcription. Their functional experiments revealed that the suppression of PVT1 had the capacity to hinder the proliferation and enhance the apoptotic processes in HLE B-3 cells. In their mechanistic inquiry, they disclosed that PVT1 operated as a 'miRNA sponge,' directing its regulatory effects towards the miR-214-3p/MMP2 axis. Their discovery has unveiled a fresh perspective on the involvement of the lncRNA PVT1/miR-214-3p/MMP2 axis in the pathogenesis of DC, offering valuable insights into the mechanisms underlying this condition (Yang et al. 2020). In addition, Guo et al. demonstrated that PVT1 exhibited increased expression in cellular models of DR, and the inhibition of PVT1 resulted in enhanced cell viability and reduced apoptosis in HG-treated ARPE-19 cells. Their findings additionally demonstrated that PVT1 could directly interact with miR-1301-3p, exerting a negative regulatory effect on miR-1301-3p expression. Subsequent experiments unveiled that miR-1301-3p directly targeted KLF7, and PVT1 elevated KLF7 expression through its interaction with miR-1301-3p. They finally confirmed that the impact on cell viability and apoptosis resulting from PVT1 silencing could be restored through KLF7 overexpression. Therefore, PVT1demonstrates a suppressive impact on the proliferation and induces apoptosis in HG-treated ARPE-19 cells by interacting with miR-1301-3p, which subsequently leads to the upregulation of KLF7 (Guo et al. 2022).

### LncRNA MIAT/miRNA axis

Zhang et al. investigated the underlying mechanism linking MIAT to DR, a crucial aspect of DR pathogenesis. They observed elevated levels of MIAT and p-p65 in streptozotocin (STZ)-induced diabetic mice and in rat retinal Müller cells (rMC-1) stimulated by HG. They observed that elevated glucose levels increased the interaction between NF-κB and MIAT. Conversely, Bay11-7082, a known inhibitor of NF-κB, attenuated this binding activity. They revealed a regulatory loop involving miR-29b and MIAT. miR-29b was found to modulate MIAT expression. In turn, upregulated MIAT functioned to inhibit miR-29b, while conversely enhancing Sp1 activity. They also established that HG stimulation elevated cellular apoptosis and reduced cellular activity. However, MIAT silencing reversed the influences induced by HG. Conversely, miR-29b silencing counteracted the effects triggered by MIAT suppression. Their findings offered evidence supporting a potential link between MIAT regulation and the mechanism of cell apoptosis in DR, with miR-29b identified as a biomarker subject to MIAT's regulatory influence on cell apoptosis in DR. Thereby, clinical treatment strategies centered on the network involving NF-κB/MIAT/miR-29b/Sp1 axis hold significance for the management of DR (Zhang et al. 2017).

### LncRNA NEAT1/miRNA axis

Li et al. explored the potential role of the lnc RNA nuclear paraspeckle assembly transcript 1 (NEAT1) in the development of DR. They demonstrated a considerable downregulation of NEAT1 in the retinas of rats with diabetes mellitus. Meanwhile, there was a notable elevation in miR-497 in the retinas of rats with diabetes mellitus and in Müller cells subjected to HG treatment. Conversely, brain-derived neurotrophic factor (BDNF) exhibited an upregulation in expression. Furthermore, their observations indicated that in vitro, Müller cells the apoptosis induced by HG was concomitant with a marked reduction in the expression of NEAT1. Subsequent investigations revealed that the induction of apoptosis in Müller cells by HG was facilitated by the downregulation of NEAT1, and reduced expression of NEAT1 exert its influence by negatively regulating miR-497. Moreover, BDNF was subject to negative regulation by miR-497 and exhibited an association with the apoptosis of Müller cells in the context of HG exposure. Thereby, in diabetic conditions characterized by NEAT1 downregulation trigger miR-497 upregulation, thereby reducing BDNF levels and exacerbating Müller cell apoptosis in, ultimately worsening DR (Li 2018). Additionally, Li et al. observed a significant reduction in NEAT1 and YY1 expression levels in the anterior capsule tissue of lenses affected by DC, and these reductions were positively correlated. Their Dual Luciferase reporter assay and ChIP analysis provided confirmation that YY1 indeed possessed the ability to bind to the second locus of NEAT1. They revealed that the downregulation of NEAT1 suppressed cell proliferation and concurrently facilitated apoptosis when exposed to HG conditions. Subsequent experiments unveiled that the depletion of NEAT1 resulted in microRNA-205-3p overexpression, and MMP16 was identified as a plausible target of miR-205. Their conclusion was that diminished YY1 expression instigates the reduction of NEAT1, which, in turn, governs the miR-205-3p/MMP16 axis, ultimately leading to apoptosis and contributing to the progression of DC (Li et al. 2020).

### LncRNA LUADT1/miRNA axis

Dai et al. via analysis of RNA interactions revealed a potential interaction between LUADT1 and miR-383. Their qPCR analysis indicated a decrease in the expression of lncRNA LUADT1 and an increase in miR-383 levels in the context of DR. As well, their study disclosed that overexpression of miR-383 and LUADT1 in RPE cells did not exert any significant influence on the expression levels of each other. While increased LUADT1 xpression correlated with an elevation of peroxiredoxin 3 (PRX3) levels, miR-383 overexpression conversely led to decreased PRX3 expression. Their analysis of cellular apoptosis demonstrated that upregulation of PRX3 and LUADT1 resulted in a decrease in apoptotic cell cell death. Conversely, miR-383 exhibited an opposing effect, mitigating the apoptotic impact of PRX3 and LUADT1 overexpression. Therefore, LUADT1 downregulation observed in DR appears to modulate the PRX3/miR-383 axis, thereby attenuating cell apoptosis and suggesting the LUADT1/miR-383/PRX3 pathway as a potential avenue for therapeutic development in DR (Dai et al. 2022).

### LncRNA XIST/miRNA axis

Wang et al. substantiated the interaction between miR-34a and both XIST and SMAD2 using a luciferase reporter assay. Their study revealed an upregulation in XIST expression and a concurrent downregulation in miR-34a levels within DC tissues and SRA01/04 cells subjected to HG treatment. They additionally showed that suppressing XIST expression or enhancing miR‑34a levels resulted in reduced cell proliferation, while inducing apoptosis in SRA01/04 cells exposed to HG conditions. It was determined that miR-34a directly interacted with SMAD2, and XIST positively regulated the expression of SMAD2. Further, in SRA01/04 cells exposed to HG, XIST silencing suppressed proliferation and enhanced apoptosis, which were however mitigated by SMAD2 upregulation. In conclusion, XIST was identified as a promoter of cell proliferation and an inhibitor of apoptosis in DC, functioning through the regulatory axis of miR‑34a/SMAD2. These findings propose the XIST/miR-34a/SMAD2 axis could serve as a potential novel biomarker for the management of DC (Wang et al. 2022a).

### LncRNA AK077216/miRNA axis

Zhang et al. revealed a notable reduction of plasma lncRNA AK077216 specifically in DR patients, as opposed to diabetic patients without evident complications, when compared to healthy controls. In their study, the reduced expression of AK077216 served as a distinguishing feature that set apart DR patients from both diabetic patients without complications and healthy controls. Furthermore, treatment with HG did not significantly affect lncRNA AK077216 expression in ARPE-19 cells. Their investigation revealed a reciprocal relationship between miR-383 and lncRNA AK077216 in DR patients, where the upregulation of lncRNA AK077216 led to a decrease in miR-383 levels. However, the upregulation of miR-383 did not induce a significant alteration in the expression of lncRNA AK077216. Additionally, their research revealed that the upregulation of lncRNA AK077216 had an inhibitory effect on apoptosis in ARPE-19 cells, whereas the upregulation of miR-383 exerted an opposing role, mitigating the impact of lncRNA AK077216 upregulation. Therefore, LncRNA AK077216 exhibits downregulation in the context of DR and exerts an inhibitory effect on apoptosis in ARPE-19 cells by means of the downregulation of miR-383 (Zhang et al. 2019).

### LncRNA SNHG16/miRNA axis

Li et al. reported elevated expression of E2F1 and SNHG16, alongside reduced miR-20a-5p levels, in proliferative DR compared to control or non-proliferative DR conditions. They discovered that SNHG16 functions as a regulatory hub, modulating miR-20a expression and its interactions with both SNHG16 and E2F1. Subsequent experiments demonstrated that overexpression of SNHG16 via plasmid transfection promoted both cell apoptosis and vessel-like formation, whereas introduction of a miR-20a-5p mimic partially abrogated these effects. They ultimately showed that the introduction of a gene silencing E2F1 plasmid effectively reversed the exacerbating effects of SNHG16 overexpression on proliferative DR. In conclusion, SNHG16 as a miR-20a-5p sponge, demonstrating its regulatory control over E2F1 expression and a consequent exacerbation of apoptosis in proliferative DR (Li et al. 2021c).

### LncRNA RPSAP52/miRNA axis

Niu et al. explored the roles of RPSAP52 in DR. Their findings indicated a notable reduction of RPSAP52 in DR patients in comparison to individuals with diabetes lacking evident complications. They revealed a direct interaction between RPSAP52 and miR‑365, but the upregulation of miR‑365 and RPSAP52 did not lead to any significant alteration in each other's expression. They also demonstrated that the RPSAP52 overexpression resulted in the upregulation of TIMP metallopeptidase inhibitor 3 (TIMP3) in RPE cells. HG treatment of RPE cells led to decreased expression of TIMP3 and RPSAP52, while miR-365 levels were conversely upregulated. Moreover, through cell apoptosis analysis, exposure to HG treatment in RPE cells resulted in a significant upregulation of both TIMP3 and RPSAP52, which was associated with a decreased rate of apoptosis. Therefore, RPSAP52 acts as an endogenous sponge for miR-365, promoting TIMP3 expression and consequently inhibiting RPE cell apoptosis in DR (Niu et al. 2020).

### LncRNA FLG-AS1/miRNA axis

Compared to healthy controls, DR patients exhibited a significant decrease in FLG-AS1 expression and a notable increase in miR-380-3p expression in their serum. Further, the expression levels of miR-380-3p, and FLG-AS1 exhibited a negative correlation among DR patients. Moreover, upregulating FLG-AS1 protected ARPE-19 cells from HG-induced apoptosis, and mitigated retinal injury in diabetic rats. Mechanistically, FLG-AS1 directly targets miR-380-3p, thereby promoting SOCS6 expression. Importantly, either suppression of miR-380-3p or upregulation of SOCS6 mitigated apoptosis in ARPE-19 cells exposed to HG conditions Thereby, FLG-AS1 acts as a protective factor in DR by regulating the miR-380-3p/SOCS6 axia, thereby mitigating apoptosis in retinal epithelial cells (Luo et al. 2022).

### LncRNA SNHG4/miRNA axis

Yu et al. elucidated the function of the lncRNA SNHG4 and its interaction with miR-200b in DR. Initially, they observed a downregulation of SNHG4 in DR. They provided evidence of a direct interaction between SNHG4 and miR-200b, with the upregulation of miR-200b not exerting any significant impact on the expression of SNHG4 in ARPE-19. In contrast, they observed that SNHG4 upregulation resulted in the increased expression of Oxr1, which is a target of miR-200b. Furthermore, their analysis of cell apoptosis revealed that miR-200b overexpression elevated the rate of apoptosis in ARPE-19 cells subjected to HG treatment. They finally substantiated that SNHG4 and Oxr1 exerted contrasting effects and counteracted the outcomes associated with the upregulation of miR-200b. In conclusion, SNHG4 potentially acts as a sponge for miR-200b, thereby restraining cell apoptosis in DR through the upregulation of Oxr1 (Yu et al. 2021).

### LncRNA MALAT1/miRNA axis

Li et al. explored the regulatory mechanisms involving MALAT1, miR-378a-3p, and PDE6g in apoptosis among RMECs exposed to HG conditions. HG induced a concomitant upregulation of PDE6G and MALAT1, while conversely inhibiting miR-378a-3p expression. Subsequent experiments revealed that MALAT1 overexpression facilitated the proliferation of RMECs and concurrently suppressed apoptosis when exposed to HG conditions. In their study, MALAT1 competitively sequestered miR-378a-3p, a molecule that targeted PDE6G. Consequently, the MALAT1/miR-378a-3p/PDE6G signaling axis functions to mitigate apoptosis in RMECs under HG conditions (Li 2021).

### LncRNA MCM3AP-AS1/miRNA axis

**Fig. 4:**
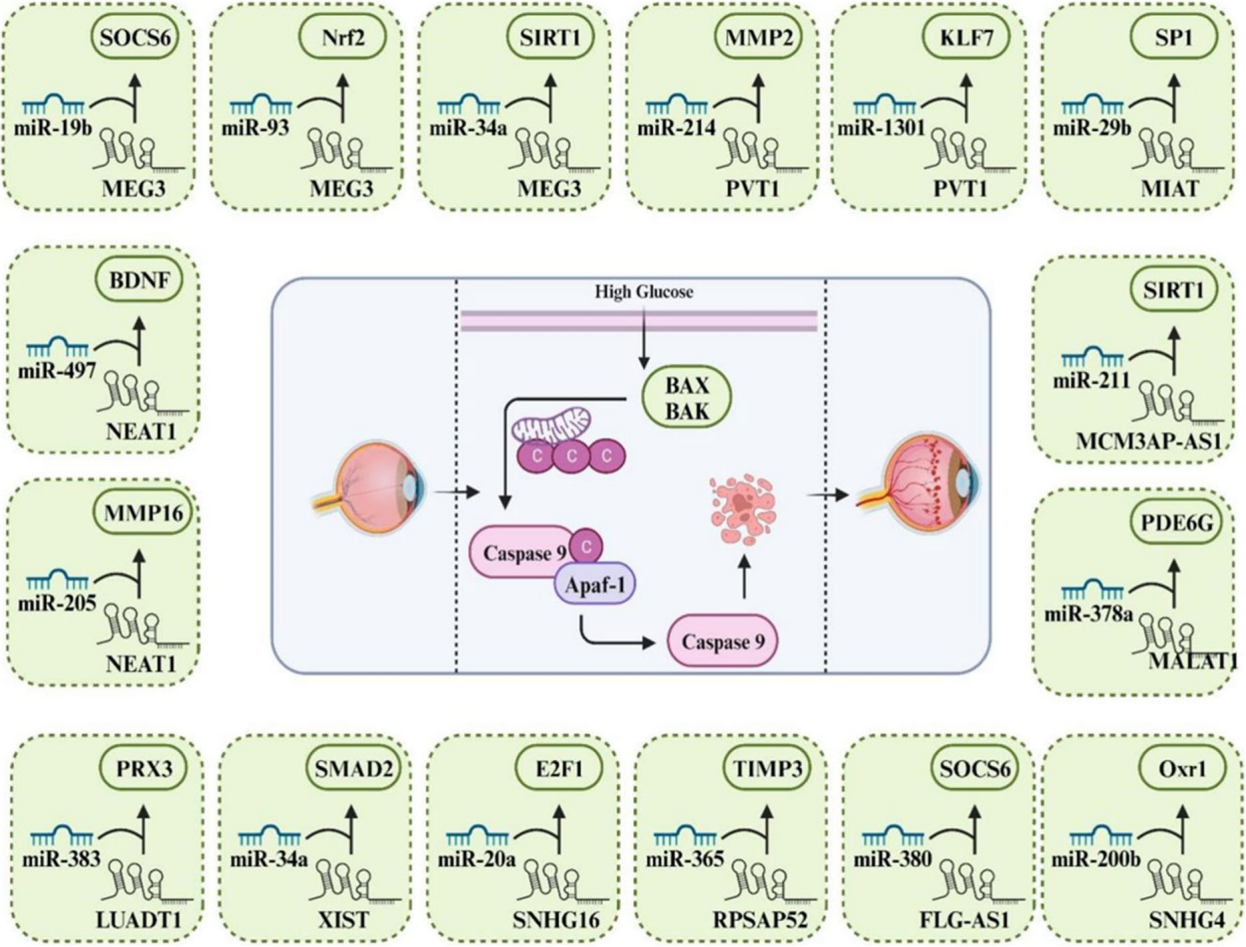
Comprehensive depiction of the intricate LncRNA/miRNA/mRNA regulatory axis orchestrating apoptosis in DR. The illustration highlights the dynamic interactions among lncRNAs, miRNAs, and mRNAs, showcasing their collective influence on apoptotic pathways within retinal cells

Recent investigation disclosed that MCM3AP-AS1 reduced in DR patients in comparison to individuals with type 2 diabetes mellitus who did not exhibit significant complications. As well, exposure to HG led to the downregulation of MCM3AP-AS1 in RPEs. Notably, according to cell apoptosis analysis overexpression of MCM3AP-AS1 and SIRT1 resulted in a decrease in the apoptotic rate of RPEs. However, the upregulation of miR-211 partially counteracted the effects of MCM3AP-AS1 and SIRT1 overexpression. In this manner, MCM3AP-AS1 exhibits reduction in DR and facilitates cell apoptosis through the regulation of the miR-211/SIRT1 pathway (Xia et al. 2022a) (Fig. 4).

## CircRNA/miRNA/mRNA axis-mediated apoptosis in DR

CircRNAs, recognized as emerging molecules of significant interest, are found in high abundance within various eukaryotic cells. They are transcribed from a substantial number of genes in both human and animals (Zhou et al. 2018). The majority of circRNAs originate from the backsplicing of exons or introns derived from pre-messenger RNA (pre-mRNA) genes responsible for encoding proteins. This process leads to the connection of the downstream 5' splicing site with the upstream 3' splicing site, resulting in the formation of a circular RNA molecule held together by a 3'-5'-phosphodiester bond (Welden and Stamm 2019). It is widely recognized that a subset of circRNAs, which contains MREs, can function as competitive molecules. These circRNAs interact with various miRNAs, thereby influencing the efficacy of miRNAs in regulating downstream mRNA expression (Su et al. 2024). Multiple investigations have provided evidence of circRNA-miRNA-mRNA regulatory pathways contributing to the apoptosis of retinal cells and the development of DR. Thereby, in the following section we will explore the current state of knowledge regarding the regulatory interactions between circRNA, miRNA, and mRNA in retinal cells apoptosis and DR.

### CircZNF532/miRNA axis

Liang et al. demonstrated that DR patients and and HG-induced ARPE-19 cells displayed upregulated STAT3 and circZNF532 expression, alongside decreased miR-20b-5p levels. They observed that increased miR-20b-5p levels or circZNF532 silencing resulted in heightened proliferation and diminished apoptosis in ARPE-19 cells. They further established that circZNF532 function as a ceRNA for miR-20b-5p, subsequently suppressing its expression. Additionally, miR-20b-5p was validated as a direct regulator of STAT3. Their findings demonstrated that miR-20b-5p directly targets STAT3, thereby regulating the viability and apoptosis of ARPE-19 cells. Their final analysis revealed elevated levels of STAT3 and circZNF532 in mice with STZ-induced diabetes, accompanied by a significant decrease in miR-20b-5p expression relative to the control group. Notably, they noted that circZNF532 silencing resulted in the suppression of apoptosis in the retinal tissues of mice. Thereby, circZNF532 overexpression may exacerbate the development of DR by undermining the suppressive action of miR-20b-5p on STAT3, offering novel insights into the pathogenesis of DR and advocating for further exploration of the circZNF532/miR-20b-5p/STAT3 axis as a promising candidate for therapeutic intervention in this condition (Liang et al. 2022).

### CircPAG1/miRNA axis

Tao et al. explored the role of circular RNA phosphoprotein associated with glycosphingolipid-enriched microdomains 1 (circPAG1) in the context of DC. In their study, the levels of circPAG1 were found to be diminished in individuals withDC, and the increased expression of circPAG1 mitigated the inhibitory effects of HG on cell viability and proliferation, while also counteracting the promotion of cell apoptosis and oxidative stress. They noted that circPAG1 acted as a miR-211-5p sponge, and the protective function of circPAG1 was partially accomplished through its sequestration of miR-211-5p. They also revealed that miR-211-5p targeted E2F3, and circPAG1 increased the expression of E2F3 by sequestering miR-211-5p. In their final analysis, they provided evidence that miR-211-5p suppression alleviated HG-induced cell apoptosis and oxidative stress by elevating E2F3 expression. In this manner, their study elucidated that circPAG1 conferred protection to human lens epithelial cells against HG-induced cell apoptosis and oxidative stress through its regulation of the miR-211-5p/E2F3 axis (Tao et al. 2022).

### CircFTO /miRNA axis

Huang et al. examined the influence of circFTO overexpression on cellular apoptosis within the context of DR. Theyrevealed miR-148a-3p reduction in DR patients, while circFTO expression correlated with increased apoptosis of ARPE-19 cells, suggesting a regulatory impact of circFTO/miR-148a-3p axis in retinal epithelial cell injury. Furthermore, silencing circFTO alleviated HG-induced injury in ARPE-19 cells, likely through suppression of oxidative stress and inflammation. In vitro studies demonstrated that circFTO modulates the expression of TGFA and miR-148a-3p. Their findings revelaed circFTO acts as a miR-148a-3p sponge, regulating TGFA expression and consequently promoting apoptosis, and DR progression. Therefore, circFTO promotes apoptosis in ARPE-19 cells, suggesting a regulatory role for the circFTO/miR-148a-3p axis in retinal epithelial cell apoptosis (Huang et al. 2022).

### Circ_NNT/miRNA axis

HG stimulation resulted in a reduction in TIMP3 and circ_NNT expression, accompanied by an elevation in miR-320b expression in ARPE-19 cells. Overexpression of circ_NNT reversed the apoptosis and inflammation induced by HG in ARPE-19 cells.Experimental studies revealed that circ_NNT functioned as a ceRNA for miR-320b, resulting in the increased expression of TIMP3. Increased miR-320b levels diminished circ_NNT protective effects on HG-induced damage to ARPE-19 cells. Furthermore, miR-320b suppression was found to protect ARPE-19 cells from HG-induced apoptosis. These protective effects were nullified by TIMP3 silencing. In summary, circ_NNT serves a protective role in ARPE-19 cells against apoptosis and inflammation induced by HG by increasing TIMP3 levels through the sequestration of miR-320b. These results imply that the circ_NNT overexpression may have a potential role in inhibiting the progression of DR (Liu et al. 2022).

### Circ_0000615/miRNA axis

**Fig. 5:**
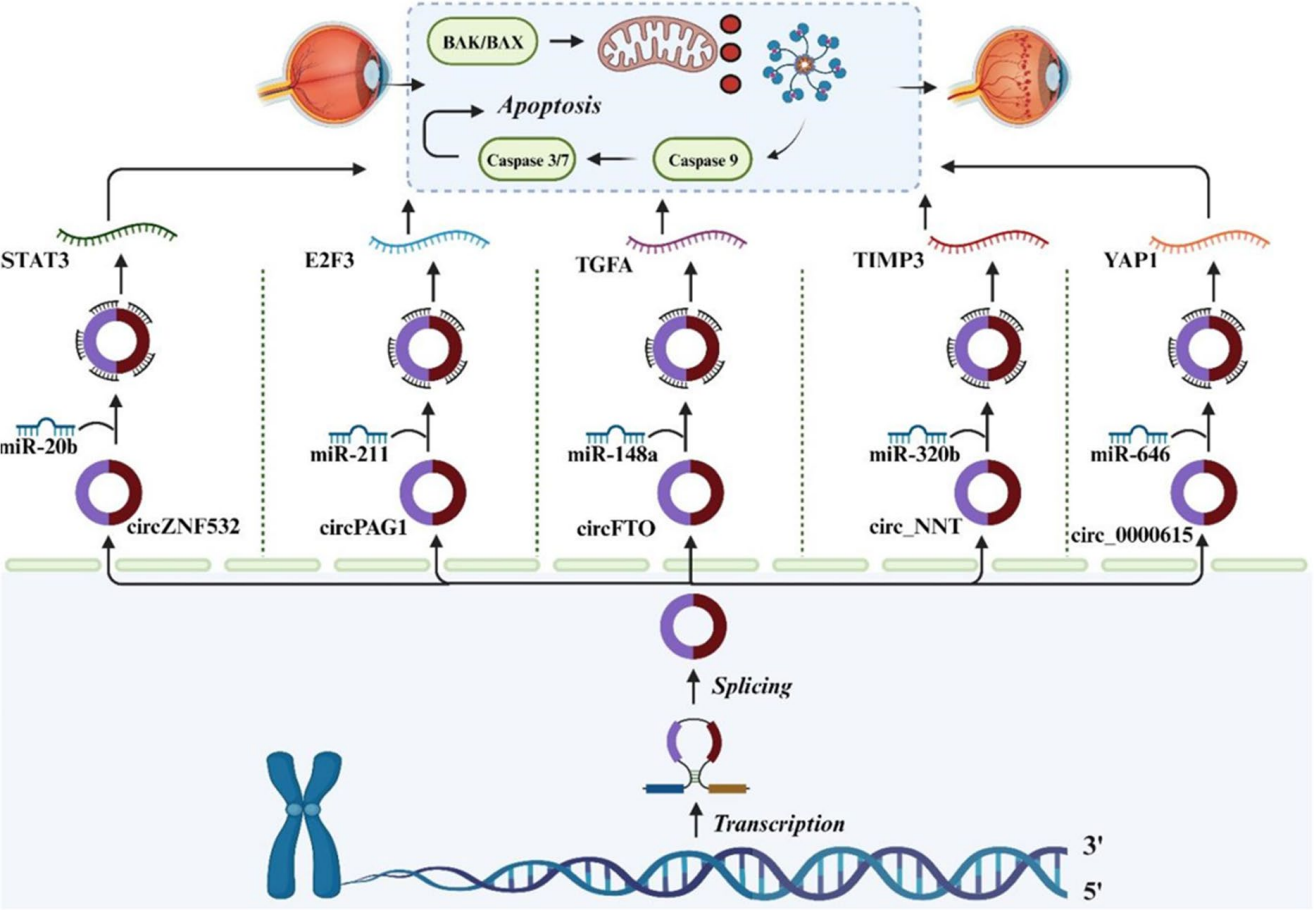
Integrated illustration unraveling the intricate CircRNA/miRNA/mRNA regulatory axis governing apoptosis in DR. The visual narrative captures the dynamic interplay among circular RNAs, microRNAs, and messenger RNAs, elucidating their collaborative impact on apoptotic pathways within retinal cells

Zeng et al. examined the specific impacts and molecular mechanisms of circ_0000615 in the progression of DR. In their study, the expression of circ_0000615 was found to be increased in HRPE cells exposed to HG. It was observed that sliencing circ_0000615 in HRPE cells exposed to HG attenuated both apoptosis and ROS production. Mechanistic investigations revealed that circ_0000615 directly targets miR-646. Moreover, inhibition of miR-646 abrogated the protective effects of circ_0000615 silencing, suggesting that circ_0000615 alleviates apoptosis in HRPE cells. As well, they confirmed that miR-646 directly targets YAP1, and the YAP1 upregulation counteracted the detrimental effects caused by miR-646 overexpression on apoptosis in HRPE cells exposed to HG. Besides that, circ_0000615 functions as a modulator of YAP1 expression via miR-646, thereby implicating the circ_0000615/miR-646/YAP1 axis in HG-induced apoptosis of HRPE cells. These findings shed new light on DR pathogenesis and highlight circ_0000615 as a promising candidate for therapeutic targeting in DR (Natesh et al. 2021) (Fig. 5).

## Targeting apoptosis in DR: from Herbal medicine to novel therapy

Emerging evidence underscores the significant contribution of apoptosis in retinal cells, particularly retinal ganglion cells, to the development of DR. This suggests the potential for apoptosis as an alternative therapeutic strategy to address DR. At present, there exist various compounds primarily focused on modulating ncRNAs to inhibit apoptosis in DN. The following section provides an overview of these strategies and agents.

### Herbal medicine

Herbal medicines are categorized as products derived from plants and employed for the purpose of maintaining or restoring health, as defined by the National Institutes of Health. Historical records indicate that herbal medicine has been employed for more than 5000 years, serving as the sole documented form of medicine during that era (Gavanji et al. 2023). Contemporary scholarly literature presents compelling evidence regarding the potential efficacy of herbal medicine as agents against DR. The active constituents within herbal medicines can interact with a variety of ncRNAs, including miRNAs, lncRNAs, and circRNAs, which are energing as promising therapeutic targets for a wide range of diseases. Thereby, these molecules, acting through ncRNAs, can modulate regulatory processes, particularly those related to pro-apoptotic pathways, and thereby offer protective effects in various pathological contexts, including diabetes. In the following section, we focus on herbal extracts that target ncRNAs and apoptosis in DR.

#### NcRNA-mediated apoptosis modulation via Astragaloside‑IV in DR

Astragaloside IV (AS-IV) is a naturally occurring saponin derived from Radix astragali, commonly known as Huangqi in traditional Chinese medicine. This compound has gained prominence for its growing recognition of possessing potential anti-apoptotic, antioxidant, and anti-inflammatory, attributes. Over the past decade, an accumulating body of evidence has underscored the protective effects of AS-IV against DM (Li et al. 2023). In a study by Wang et al., AS-IV protective effects on RPE cells in diabetic rats were investigated, along with the associated molecular mechanisms. AS-IV reduced apoptosis in RPE cells from diabetic rats, downregulated protein expressions of apoptosis pathways related factors, including active caspase‑8, active caspase-9, active caspase-3, Fas/FasL, Bax/Bcl-2, p-p70S6K1/p70S6K1, p-AKT/AKT, HOXB3, and PI3K, while simultaneously upregulating miR-128 expression.They also demonstrated that the administration of AS-IV successfully suppressed apoptosis induced by HG in RPE cells by increasing the expression of miR-128 and the levels of Bcl-2 and FasL proteins. Altogether, treatment with AIV-IV protects RPE cells in diabetic rats from apoptosis, protentially through the increased expression of miR-128 (Wang et al. 2020b).

#### NcRNA-mediated apoptosis modulation via Astragalus polysaccharides in DR

Astragalus membranaceus, a renowned traditional herbal remedy, has enjoyed extensive usage in treating various ailments for over 2000 years. The primary bioactive constituents derived from A. membranaceus, such as flavonoids, triterpene saponins, and polysaccharides, have exhibited a diverse array of biological properties and pharmacological actions. Astragalus polysaccharide (APS), a prominent bioactive constituent of Astragalus membranaceus, has been the subject of extensive research due to its pharmacological properties, which encompass antidiabetic, neuroprotective, and antiapoptotic effects (Wang et al. 2023). Peng et al.observed that exposure HG levels induced metabolic memory, characterized by persistent aberrations in the miR-204/SIRT1 axis, elevated endoplasmic reticulum (ER) stress, and apoptotic pathways activation, even after switching back to normal glucose levels. Notably, a concentration-dependent increase in APS treatment reversed miR-204 expression, leading to SIRT1 derepression and subsequent mitigation of ER stress-induced apoptosis. This was evidenced by reduced levels of cleaved caspase-12, -9, -3, Bax, cleaved-ATF6, p-PERK, and p-IRE-1, along with increased levels of Bcl-2 and unprocessed PARP. As well, the effects of APS on RPE cells were counteracted when miR-204 was upregulated or SIRT1 was silenced. Therefore, in a metabolic memory model of RPE cells, APS treatment alleviated ER stress and subsequent apoptosis by regulating the miR-204/SIRT1 axis, suggesting its therapeutic potential for DR (Peng et al. 2020). Similarly, investigating the potential reversal of metabolic memory in RPE cells, Liu et al. examined the effects of APS following exposure to HG. In their study, the administration of APS resulted in a notable reduction in the levels of miR-195 and an elevation in Bcl-2 expression, even in cases where HG treatment was replaced with normal glucose (NG), and this effect was dose-dependent. They recognized Bcl-2 as a direct miR-195 target and further revealed that APS mitigated oxidative stress, mitochondrial impairment, and cellular apoptosis induced by both HG and a combination of HG and NG (HG + NG) treatments in RPE cells by modulating miR-195. They finally found that beneficial effects of APS on RPE cells subjected to HG conditions were negated by miR-195 upregulation. In this manner, APS effectively mitigated the metabolic memory phenomenon in RPE cells exposed to HG by preventing mitochondrial dysfunction-induced apoptosis through the regulation of miR-195 (Liu et al. 2019b). Moreover, Gao et al. utilized ARPE-19 cell lines and primary RPE cells to empirically assess the impact of APS on mitochondrial damage and apoptosis triggered by metabolic memory resulting from HG exposure. They observed that a regimen of HG followed by NG exposure significantly elevated the levels of miR-182 and concurrently reduced the expression of its target gene, Bcl-2. APS treatment effectively counteracted these aforementioned effects. They additionally noted that APS treatment reinstated proper mitochondrial function and mitigated apoptosis in cells exhibiting metabolic memory. In their final observation, they found that the protective effects of APS against mitochondrial damage and apoptosis were partially diminished upon upregulation of miR-182. In this manner, APS mitigated mitochondrial damage and apoptosis induced by metabolic memory through the regulation of the miR-182/Bcl-2 axis, potentially offering a novel therapeutic approach for managing DR (Gao et al. 2021).

#### NcRNA-mediated apoptosis modulation via blueberry anthocyanin extract in DR

Blueberries are recognized as a nutritious fruit due to their high anthocyanin content. An increasing body of research indicates that anthocyanins derived from the berries of blueberry plants offer significant nutritional and health benefits. Beyond their widely recognized antioxidant properties, the prominent active constituents in blueberries, specifically anthocyanins, exhibit notable anticancer and anti-type 2 diabetes properties (Wu et al. 2023). Wang et al. observed that BAE effectively suppressed ROS production, ERS, and apoptosis in RP cells induced by HG (H-Glu). Their experiments revealed that the upregulation of miR-182 expression caused by H-Glu was reversed by BAE, suggesting that miR-182 might serve as an intermediary molecule through which BAE mitigates H-Glu-induced stress damage in ARPE-19 cells. Their mechanistic investigation uncovered that miR-182 directly interacted with OGG1, leading to the induction of ROS generation, and consequently resulting in increased levels of ERS and apoptosis in H-Glu-treated ARPE-19 cells. In this manner, their findings strongly indicate that in RPE cells, BAE mitigates ER stress by modulating the miR-182/OGG1 axis, thereby reducing apoptosis induced by HG (Wang et al. 2022b).

#### NcRNA-mediated apoptosis modulation via resveratrol in DR

Resveratrol (RSV), also known as 3,5,4'-trihydroxystilbene, is a non-flavonoid polyphenol that is naturally produced as a phytoalexin. It is synthesized by various plant sources, including peanuts, plums, blueberries, grapes, and apples. This compound plays a significant role in human health and is widely recognized for its various biological functions, including anti-inflammatory and antioxidant properties (Meng et al. 2023). Zeng et al. disclosed that TUNEL-positive cells were predominantly situated within the inner nuclear layer (INL) of the retina. Administration of RSV exhibited a notable suppression of streptozotocin (STZ)-induced apoptosis in retinal cells within the INL. Notably, diminished expression of miR-29b and the heightened expression of SP1 induced by diabetes were found to be effectively restored by RSV, both in vivo and in vitro. As well, anti-apoptotic impact of RSV and its ability to decrease SP1 expression were hindered when a miR-29b inhibitor was introduced. Therefore, RSV holds promise as a potential therapeutic intervention for DR, and it appears that the miR-29b/SP1 pathway is involved in the anti-apoptotic mechanism of RSV (Zeng et al. 2017b).

#### NcRNA-mediated apoptosis modulation via Dihydromyricetin in DR

Extracted from Ampelopsis grossedentata, Dihydromyricetin (DHM), a naturally occurring flavanol compound, exhibit diverse pharmacological properties, including antitumor, anti-inflammatory, and antioxidative effects. Studies by Li et al. revealed a dose-dependent increase in cell viability upon DHM treatment in cells exposed to HG. In their study, the concentration of ROS exhibited a notable decrease in ARPE-19 cells exposed to HG stimulation. Furthermore, DHM treatment significantly attenuated HG-induced apoptosis in ARPE-19 cells. Moreover, exposure to HG resulted in a marked upregulation of miR-34a expression, which was subsequently reversed upon DHM treatment. Furthermore, the ability of DHM to suppress HG-induced apoptosis in ARPE-19 cells was abolished by miR-34a upregulation. In this manner, DHM demonstrates protective properties against apoptosis in DR by suppressing the expression of miR-34a, warrants further exploration for its therapeuticpotential in DR (Li and Xiao 2021).

### Novel therapy in DR

**Fig. 6:**
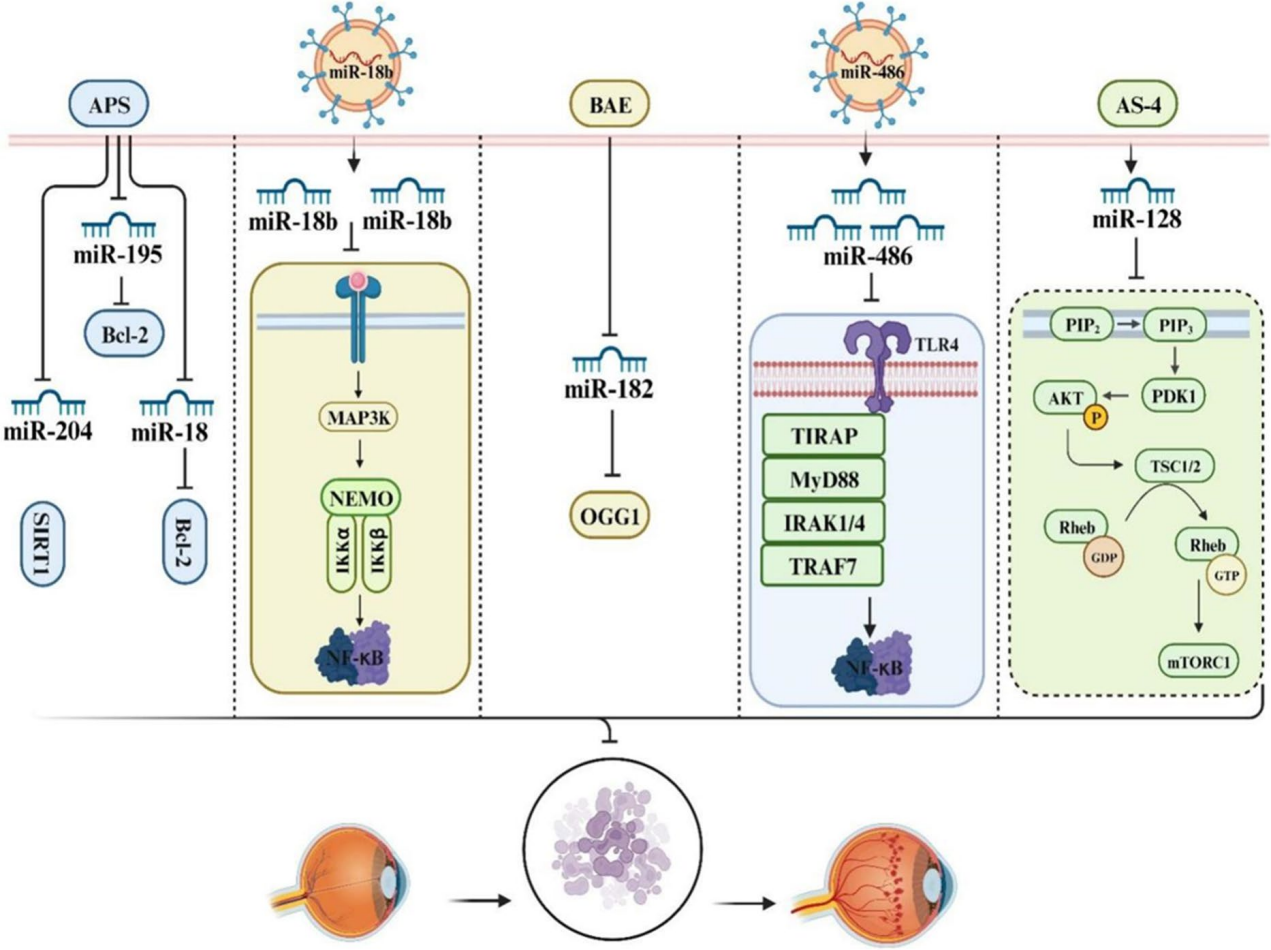
Comprehensive depiction of therapeutic strategies targeting apoptosis in DR, spanning from traditional herbal medicines (BAE, AS-4, APS BY) to innovative exosomal miRNA-based interventions. The visual narrative elucidates the antiapoptotic effects of herbal remedies (BAE, AS-4, APS BY), showcasing their modulation of miRNA networks within retinal cells. Additionally, the figure underscores the emerging role of exosomal miRNAs in conferring anti-apoptotic benefits

For a long time, it has been documented that cells release vesicles into the extracellular space. These extracellular vesicles (EVs) come in various forms, which are distinguished by their size and the mechanisms underlying their formation. There exist three recognized categories of EVs: microvesicles, apoptotic bodies, and exosomes. Among these, exosomes are released by a wide range of cell types. Exosomes are involved in both normal and pathological cellular interactions by facilitating the transfer of RNA, lipids, and proteins. The composition and impact of exosomes are contingent on the characteristics of the donor cell. Exosomes released by specific cell types, such as stem cells, transport bioactive molecules associated with the protection, regeneration, and promotion of blood vessel formation in injured tissues. Consistent with these findings, Li et al. explored the functional significance of exosomal miR-17–3 p in modulating apoptosis in a mice model of DR. They firstly generated a diabetic mouse model. Following this, exosomes derived from human umbilical cord mesenchymal stem cells (hucMSCs) were isolated and enriched with miR-17-3p before being administered to the mice. A reduction in retinal miR-17-3p expression was observed in DR mice. Conversely, STAT1 expression was increased, suggesting that STAT1 may be a direct target miR-17-3p. Exosome administration enriched with miR-17-3p improved metabolic parameters (reduced blood glucose and HbA1c levels, increased body weight, hemoglobin content, and GS levels) in DR mice. Additionally, it dampened inflammation and vascular dysfunction (decreased inflammatory factors and VEGF levels), mitigated oxidative damage, and suppressed retinal cell apoptosis. These effects were achieved through the inhibition of STAT1 by miR-17-3p delivered via exosomes. In this manner, hucMSCs-derived exosomes deliver miR-17-3p to suppress inflammatory responses and apoptosis in DR mice, acting through the downregulation of STAT1 (Li et al. 2021). Furthermore, Li et al. sought to elucidate the mechanisms underlying the impact of BMSCs-derived exosomal miR-486-3p in DR. In their study, miR-486-3p exhibited low expression levels, whereas NF-κB and TLR4 exhibited elevated expression levels in HG-exposed Müller cells. They also identified a direct interaction between miR-486-3p and TLR4. The results suggested that increasing miR-486-3p expression or decreasing TLR4 levels resulted in diminished apoptosis, while enhancing the proliferation in Müller cells subjected to HG conditions. Further experiments revealed that BMSC-derived exosomes had suppressive activities in inflammation, oxidative stress, and apoptosis, while promoting the proliferation of HG-exposed Müller cells. Additionally, they noted that reintroduction of miR-486-3p increased the beneficial effects of exosome therapy, whereas the upregulation of TLR4 counteracted these beneficial outcomes. In summary, their research has elucidated the mechanism through which exosomal miR-486-3p derived from BMSCs enhances retinal cell viability and mitigates apoptosis by inhibiting the TLR4/NF-κB pathway. Their research has the potential to broaden our understanding of potential treatments for DR by exploring targeted therapies based on miR-486-3p/TLR4/NF-κB pathways (Li et al. 2021d). In addition, Xu et al. explored the anti-apoptotic characteristics of small extracellular vesicles (sEVs) derived from human umbilical cord mesenchymal stem cells (hUCMSCs). Their investigation employed two models: human retinal microvascular endothelial cells and a diabetic rat mode. Their findings indicated that administering hUCMSC-sEVs to the retinas of diabetic rats resulted in a reduction in vascular leakage, reduced retinal thickness, and related inflammation. hUCMSC-sEVs also inhibited cell inflammation and apoptosis induced by HG in in vivo settings. Subsequently, they performed miRNA profiling in hUCMSC-sEVs and predicted miR-18b targets. Furthermore, sEV treatment induced a significant upregulation of miR-18b expression in the retinas of diabetic rats Their ultimate discovery revealed that miR-18b targets MAP3K1, thus preventing the phosphorylation of NF-κB p65 and ameliorating DR. Overall, these findings underscores the potential utility of hUCMSCs-sEVs as biomaterials for mitigating inflammation and apoptosis in DR through the transfer of miR-18b (Xu et al. 2021). These discoveries elucidate the functions and mechanisms of SC-Exos-miRNAs in the management of diabetes and associated complications, including DR (Fig. 6).

## Conclusion

**Fig. 7:**
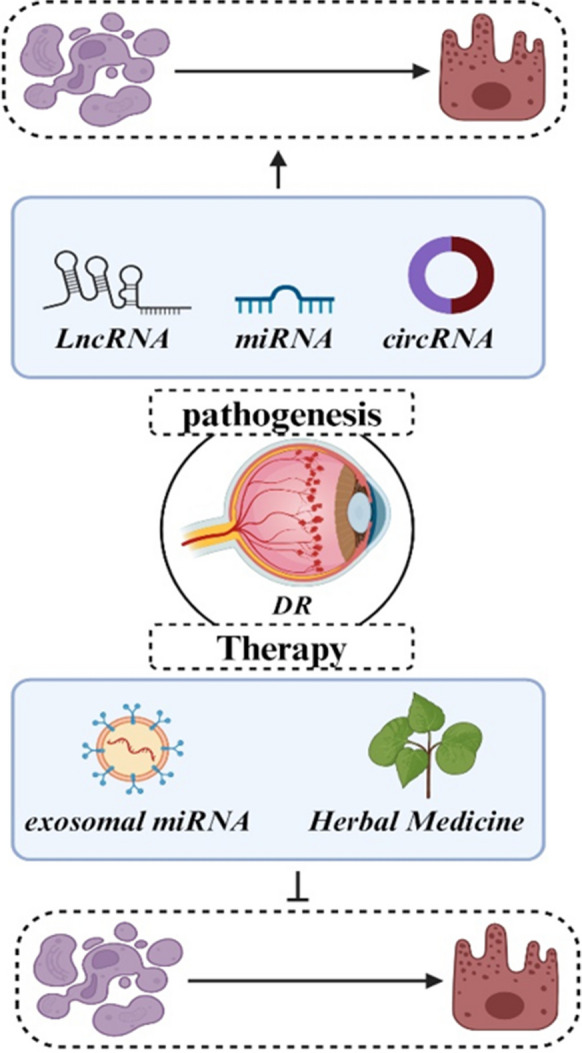
Schematic representation illustrating the interplay between non-coding RNAs and apoptosis in diabetic retinopathy. Dysregulation of non-coding RNAs promotes retinal cell apoptosis (top), while interventions such as herbal medicine and exosomal miRNAs inhibit retinal cell apoptosis (bottom), highlighting potential therapeutic strategies

Diabetes mellitus is a prevalent medical condition that has become more common in recent decades, presenting a significant public health challenge in the twenty-first century. DM serves as a primary contributor to the development of DR, a diabetes complication characterized by the obstruction of ocular blood vessels due to elevated glucose levels, leading to inflammation and leakage of blood or fluids, ultimately resulting in significant ocular damage. The development of DR may involve apoptotic cell death in retinal cells as part of its pathogenesis. Here, our research has shown that key signaling pathways such as PI3K/AKT/mTOR, TLR4/NF-κB, and SIRT1 play significant roles in the apoptosis of retinal cells and the pathogenesis of DR. Notably, an increasing number of research has revealed that ncRNAs can regulate apoptosis in renal cells through various mechanisms. In this context, a recent study has shown that suppressing miR-93 can reduce cell apoptosis and ROS levels in the retinas of DR rats by increasing Nrf2 expression and body's anti-oxidative stress levels (Yin et al. 2019). As well, a recent study has suggested that suppressing miR-93 reduces cell apoptosis and ROS levels in the retinas of DR rats by enhancing the expression of Nrf2 and increasing the body's anti-oxidative stress levels. Furthermore, overexpression of miR-20b-3p, through targeting TXNIP, has been shown to improve visual function and mitigate inflammatory responses, cell apoptosis, vascular permeability, microvascular damage, and angiogenesis in DR rats. These effects contribute to the mitigation of DR progression. These findings suggest miR-20b-3p/TXNIP axis as a novel therapeutic possibilities for DR management (Wang et al. 2020c). Notably, multiple lncRNAs and circRNAs such as MALAT1, SNHG4, FLG-AS1, SNHG16, circ-0000615, circ-NNT, circFTO, circPAG1, and so forth functions as ceRNAs, targeting microRNAs and influencing the retinal cells apoptosis in DR. Additionally, our findings in the final section reveal that ncRNA-mediated apoptosis modulation via herbal medicine and stem cells-derived exosomes could be exploited to prevent and manage DR (Fig. 7).

## Data Availability

All data supporting the findings of this study are available within the paper.

## Abbreviations

3'-UTR: 3'-Untranslated region
4-HNE: 4-Hydroxynonenal
APS: Astragalus polysaccharide
AS-IV: Astragaloside IV
Atf1: Activating transcription factor 1
BAE: Blueberry anthocyanin extract
BBR: Berberine
BDNF: Brain-derived neurotrophic factor
CARM1: Co-activator-associated arginine methyltransferase 1
CAT: Catalase
ceRNAs: Competing endogenous RNAs
circPAG1: Circular RNA phosphoprotein associated with glycosphingolipid-enriched microdomains 1
circRNAs: Circular RNAs
DC: Diabetic cataract
DHM: Dihydromyricetin
dKO: Knockout
DOK5: Docking protein 5
DR: Diabetic retinopathy
ERK: Extracellular-signal-regulated kinases
EVs: Extracellular vesicles
GNAI2: G protein subunit alpha i2
HG: High glucose
hucMSCs: Human umbilical cord mesenchymal stem cells
INL: Inner nuclear layer
JNK: Jun N-terminal kinase
lncRNAs: Long non-coding RNAs
LRH-1: Liver receptor homolog-1
MAPKs: Mitogen-activated protein kinases
miRISC: MiRNA-induced silencing complex
miRNAs: MicroRNAs
MnSOD: Manganese superoxide dismutase
MREs: MiRNA response elements
MyD88: Myeloid differential factor 88
ncRNAs: Non-coding RNAs
NEAT1: Nuclear paraspeckle assembly transcript 1
NG: Normal glucose
Oxr1: Oxidation resistance 1
PDLIM1: PDZ and LIM domain protein 1
PRDX3: Peroxiredoxin 3
PRRs: Pattern recognition receptors
PRX3: Peroxiredoxin 3
RGCs: Retinal ganglion cells
RMECs: Retinal microvascular endothelial cells
RhoA: Ras homolog family member A
RRPs: Rat retinal pericytes
RSV: Resveratrol
sEVs: Small extracellular vesicles
SGCZ: Sarcoglycan zeta
SNHG16: Small nucleolar RNA host gene 1
SIRT1: Sirtuin 1
SOCS3: Suppressor of cytokine signaling 3
STAT1: Signal transducer and activator of transcription 1
STZ: Streptozotocin
TGF-β2: Transforming growth factor-β2
TIMP3: Tissue inhibitor of metalloproteinases-3
TRIF: Toll/interleukin 1 receptor domain-containing adaptor inducing interferon-beta
TLR4: Toll-like receptor 4
TLRs: Toll-like receptors
TRPM3: Transient receptor potential melastatin 3
WT: Wild-type
XIST: X-inactive specific transcript
